# A Novel Necroptosis-Related lncRNA Signature for Predicting Prognosis and Immune Response of Glioma

**DOI:** 10.1155/2022/3742447

**Published:** 2022-06-16

**Authors:** Zhikang Wu, Meimei Liu, Jinlong Fu, Jinwei Li, Lingyao Qin, Liuying Wu, Hongmou Chen, Xianlei Yan, Quan Liu, Jiemin Zheng

**Affiliations:** ^1^Department of Neurosurgery, The Fourth Affiliated Hospital of Guangxi Medical University, Liuzhou, China; ^2^Faculty of Life Science, Hubei University, Wuhan, China

## Abstract

Glioma is one of the most common intracranial malignancies that plagues people around the world. Despite current improvements in treatment, the prognosis of glioma is often unsatisfactory. Necroptosis is a form of programmed cell death. As research progresses, the role of necroptosis in tumors has gradually attracted the attention of researchers. And lncRNA is regarded as a critical role in the development of cancer. Therefore, this study is aimed at establishing a prognostic model based on necroptosis-associated lncRNAs to accurately assess the prognosis and immune response of patients with glioma. The RNA sequences of glioma patients and normal brain samples were downloaded from The Cancer Genome Atlas (TCGA) and GTEx databases, respectively. The coexpression analysis was performed to identify the necroptosis-related lncRNAs. Then, we utilized LASSO analysis following univariate Cox analysis to construct a prognostic model. Subsequently, we applied the Kaplan-Meier curve, time-dependent receiver operating characteristics (ROC), and univariate and multivariate Cox regression analyses to assess the effectiveness of this model. And the functional enrichment analyses and immune-related analyses were employed to investigate the potential biological functions. A validation set was obtained from the Chinese Glioma Genome Atlas (CGGA) database. And qRT-PCR was employed to further validate the expression levels of selected necroptosis-associated lncRNAs. Seven necroptosis-related lncRNAs (FAM13A-AS1, JMJD1C-AS1, LBX2-AS1, ZBTB20-AS4, HAR1A, SNHG14, and LINC00900) were determined to construct a prognostic model. The area under the ROC curve (AUC) was 0.871, 0.901, and 0.911 at 1, 2, and 3 years, respectively. The risk score was shown to be an important independent predictor in both univariate and multivariate Cox regression analyses. Through functional enrichment analyses, we found that the differentially expressed genes (DEGs) were mainly enriched in protein binding and signaling-related biological functions and immune-associated pathways. In conclusion, we established and validated a novel necroptosis-related lncRNA signature, which could accurately predict the overall survival of glioma patients and serve as potential therapeutic targets.

## 1. Introduction

Glioma is the most common type of primary intracranial malignancy, accounting for approximately 84% of all malignant brain tumors (1). Traditional treatments of glioma are mainly based on surgery, supplemented by radiotherapy and chemotherapy, but the outcomes of patients are often poor (2). The five-year survival rate of high-grade glioma (HGG) patients is less than 5% (3). Although the prognosis of patients with low-grade glioma (LGG) is better than that of glioblastoma (GBM), they often face the risk of recurrence and transition to HGG (4, 5). Therefore, new treatment strategies to handle this enormous burden are urgently needed.

Necroptosis is a novel type of programmed cell death mediated mainly through the activation of the RIPK1-RIPK3-MLKL signaling pathway by the TNFR superfamily, which ultimately leads to plasma membrane rupture, lysis, and the release of inflammatory factors (6, 7). Among the mechanisms of drug resistance in tumors, the evasion of and resistance to apoptosis are considered to be the most important causes and often lead to the failure of traditional chemotherapy regimens (8). In contrast, related studies have shown that some conventional proapoptotic chemotherapeutic agents can bypass the resistance mechanism and exert antitumor effects by inducing necroptosis (9, 10). This plays an extremely significant role in the death of tumor cells. Moreover, some reports have suggested that necroptosis plays a key role in limiting cancer metastasis, for example, shikonin could reduce osteosarcoma metastasis through induction of necroptosis (11, 12).

Long noncoding RNAs (lncRNAs) have extremely important biological functions and play critical roles in cell cycling, proliferation, immune response, transcription, and translation (13–15). There are increasing evidences that lncRNAs are closely related to tumor development. For instance, HOTAIR is overexpressed in various cancers and correlates with the metastasis and invasion of these cancers (16). MALAT1 has been determined to be an important potential therapeutic target in lung adenocarcinoma and, in addition, it has a role in tumor growth inhibition in patients with glioma (17, 18). Therefore, developing new lncRNA-related therapeutic strategies and relevant tumor subtypes seem to be of great practical importance.

Our study is aimed at creating a necroptosis-associated lncRNA model to predict the prognosis of patients with glioma and to explore the immune status and tumor microenvironment of patients in different subtypes to provide new insights for individualized treatment of glioma.

## 2. Materials and Methods

### 2.1. Acquisition and Collation of Raw Data

The RNA sequences (FPKM value) and matching clinical data of 698 glioma patients (project: TCGA-GBM, TCGA-LGG; Disease Type: gliomas) were obtained from TCGA database (https://portal.gdc.cancer.gov). The RNA expression data of 1152 normal brain samples were downloaded from the GTEx database (https://gtexportal.org/home/datasets). We combined TCGA and GTEx data and then performed the “normalizeBetweenArrays” algorithm of the “limma” package to eliminate the batch effects. And the genetic mutation files of GBM and LGG patients were downloaded from TCGA database. Clinical and transcriptome data of 1018 glioma samples were achieved from the CGGA database (http://www.cgga.org.cn/) for external validation. We then excluded patients with unknown clinical features. Finally, the training and testing cohorts were constructed after integration and standardization.

### 2.2. Screening of Differentially Expressed Necroptosis-Related lncRNAs

We captured 67 necroptosis-related genes from previous studies and listed them in Table S1 (19–21). Firstly, we applied the “maftools” package to generate mutation waterfall plots for LGG and GBM separately. Then, we downloaded the human gene annotation file from the GENCODE website (https://www.gencodegenes.org). Through the “gene biotype” in the file, we can know which gene belongs to protein-coding RNA and which gene belongs to long noncoding RNA. We used a Perl script to extract the lncRNA matrix from the gene expression matrix of the training set based on the “gene biotype” in the annotation file. And finally, a total of 732 lncRNAs were screened out. Next, we performed correlation analysis to determine the necroptosis-related lncRNAs with the criterion of correlation coefficients > 0.6 and *p* < 0.001 (Table S2). And the “limma” package was utilized to identify the differentially expressed lncRNAs between normal and tumor tissues (FDR < 0.05 and |log2FC| ≥ 1). Finally, we took the intersection of the two results to obtain the differentially expressed necroptosis-associated lncRNAs and then listed them in Table S3.

### 2.3. Construction and Validation of a Prognostic lncRNA Signature

The univariate Cox analysis was conducted to identify the differentially expressed necroptosis-associated lncRNAs with prognostic significance, and the results were placed in Table S4. Then, we utilized the “glmnet” package to perform LASSO regression of these lncRNAs. For minimizing the deviations, we used the “cv.glmnet” function to choose the best lambda. And eventually, the prognostic signature was established. Each glioma patient was assigned an individual risk score, and the formula for the risk score was defined as follows (where *n*, exp(lncRNA^*i*^), and coef(lncRNA^*i*^) represented the quantity of those screened lncRNAs, the relevant expression level of each candidate lncRNA, and the regression coefficient of each lncRNA, respectively):

(1) $$\begin{array}{c} \text{risk score}=\overset{n}{\underset{i=1}{\sum }}\exp\left({\text{lncRNA}}^{i}\right)*\text{coef}\left({\text{lncRNA}}^{i}\right). \end{array}$$

For predicting the accuracy of the prognostic model, the “timeROC” package was employed to do the ROC analysis. And we utilized the “ggplot2” and “Rtsne” packages to perform the principal component analysis (PCA) and *t*-distributed stochastic neighbor embedding (t-SNE) analysis. Then, univariate and multivariate Cox regression analyses were applied to assess whether the risk score could be used as an independent predictor of prognosis. Finally, we used the “rms” package to integrate the risk score, gender, age, and tumor grade to draw a nomogram and applied calibration curves to assess whether the prediction results matched the actual ones.

### 2.4. GO and KEGG Enrichment Analyses

The Kyoto Encyclopedia of Genes and Genomes (KEGG) and Gene Ontology (GO) enrichment analyses were conducted using the Sangerbox tools, a free online platform for data analysis (http://www.sangerbox.com/tool). And the criterion was *p* < 0.05 and FDR < 0.1.

### 2.5. Immune-Related Analyses

The single-sample gene set enrichment analysis (ssGSEA) was carried out using the “GSVA” and “limma” R packages, and then, we calculated the infiltration scores of 16 immune cells and analyzed the activity of 13 immune-related pathways based on ssGSEA. Furthermore, we utilized the “limma” package to compare the expression levels of immune checkpoints in glioma patients from the high- and low-risk subgroups.

### 2.6. Exploration of Tumor Microenvironment

For investigating the differences in tumor microenvironment between the high- and low-risk categories, we utilized the “estimate” R package to score each glioma patient. And then, we visualized the results with the “ggpubr” package.

### 2.7. Quantitative Real-Time Polymerase Chain Reaction of Glioma Tissues and Matched Normal Brain Tissues

All tissues were collected from the Department of Neurosurgery of Liuzhou Workers' Hospital and approved by the hospital's medical ethics committee. Each patient has signed the relevant informed consent. We first extracted total RNA from glioma tissues and normal brain tissues using the Animal Total RNA Isolation Kit (Invitrogen, Beyotime, Shanghai, China) and then reversed the RNA into cDNA using the Transcription First-Strand cDNA Synthesis Kit (Beyotime, Shanghai, China). Finally, the quantitative real-time polymerase chain reaction (qRT-PCR) analysis was performed using BeyoFast™ SYBR Green (Beyotime, Shanghai, China). All expression data were calculated based on the 2-*ΔΔ*Ct method, and we used GAPDH as an internal control. The primers used in this study were as follows: FAM13A-AS1 forward primer, 5′-CCTGTGTGGGTCTCCATTCT-3′; FAM13A-AS1 reverse primer, 5′-TCCTCACGTGTGTGAAAGGT-3′; JMJD1C-AS1 forward primer, 5′-GGGAACCGATGAAACCTCAC-3′; JMJD1C-AS1 reverse primer, 5′-CTGGGAGTCCAAAGGAGTGT-3′; LBX2-AS1 forward primer, 5′-GGCATGGCATACAGACAAGG-3′; LBX2-AS1 reverse primer, 5′-GCAAGGGCAACTTCAAGGAA-3′; ZBTB20-AS4 forward primer, 5′-CCGTAATCCCAGCACTTTGG-3′; ZBTB20-AS4 reverse primer, 5′-GGTCTCGAACTCCTGACCTT-3′; HAR1A forward primer, 5′-GACAGAAGATGGGCGTTCCA-3′; HAR1A reverse primer, 5′-TGCCAGGTGTGAGATTGACC-3′; SNHG14 forward primer, 5′-CTTTTTCCCCTGCAATGCGT-3′; SNHG14 reverse primer, 5′-CCCCCGGGTCATGAAAACAT-3′; LINC00900 forward primer, 5′-ACTGTGCTTCTGATGACCCG-3′; LINC00900 reverse primer, 5′-ATCAGTGTCAGCGTTGAGGG-3′; GAPDH forward primer, 5′-CCGCATCTTCTTGTGCAGTG-3′; GAPDH reverse primer, 5′-TCCCGTTGATGACCAGCTTC-3′.

## 3. Results

### 3.1. Genetic Mutation and Necroptosis-Associated lncRNA Screening

**Figure 1:**
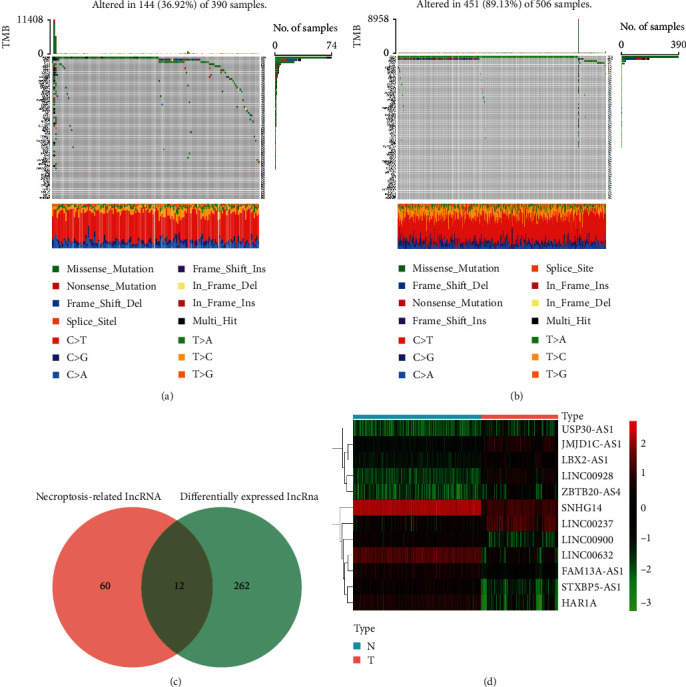
Genetic mutation diagrams and identification of necroptosis-related lncRNAs in the training cohort. (a, b) The genetic mutation landscapes of necroptosis-related genes in GBM and LGG patients. (c) The result of 12 differentially expressed necroptosis-associated lncRNAs. (d) Heatmap of the differences in 12 lncRNAs between the tumor and normal individuals.

The genetic mutation diagrams of necroptosis-related genes in GBM and LGG were shown in Figures 1(a) and 1(b). We found that 36.92% (144/390) of GBM patients had genetic mutations in necroptosis-associated genes. The highest mutation frequency among these genes was in EGFR, followed by ATRX and IDH1. While, in LGG patients, mutations were found in 89.13% (451/506) of the samples, the highest mutation rate was in IDH1, followed by ATRX and EGFR. Missense mutation was the most common type of variant in both GBM and LGG samples, and C>T was the most common in the SNV class. And as shown in Figure 1(c), we identified 12 differentially expressed necroptosis-associated lncRNAs between normal and tumor samples. We then presented a heatmap to demonstrate the differential expression between the two categories in Figure 1(d). As the heatmap showed, 6 lncRNAs were upregulated and 6 lncRNAs were downregulated in the tumor category.

### 3.2. Construction and Validation of the Prognostic lncRNA Signature

**Figure 2:**
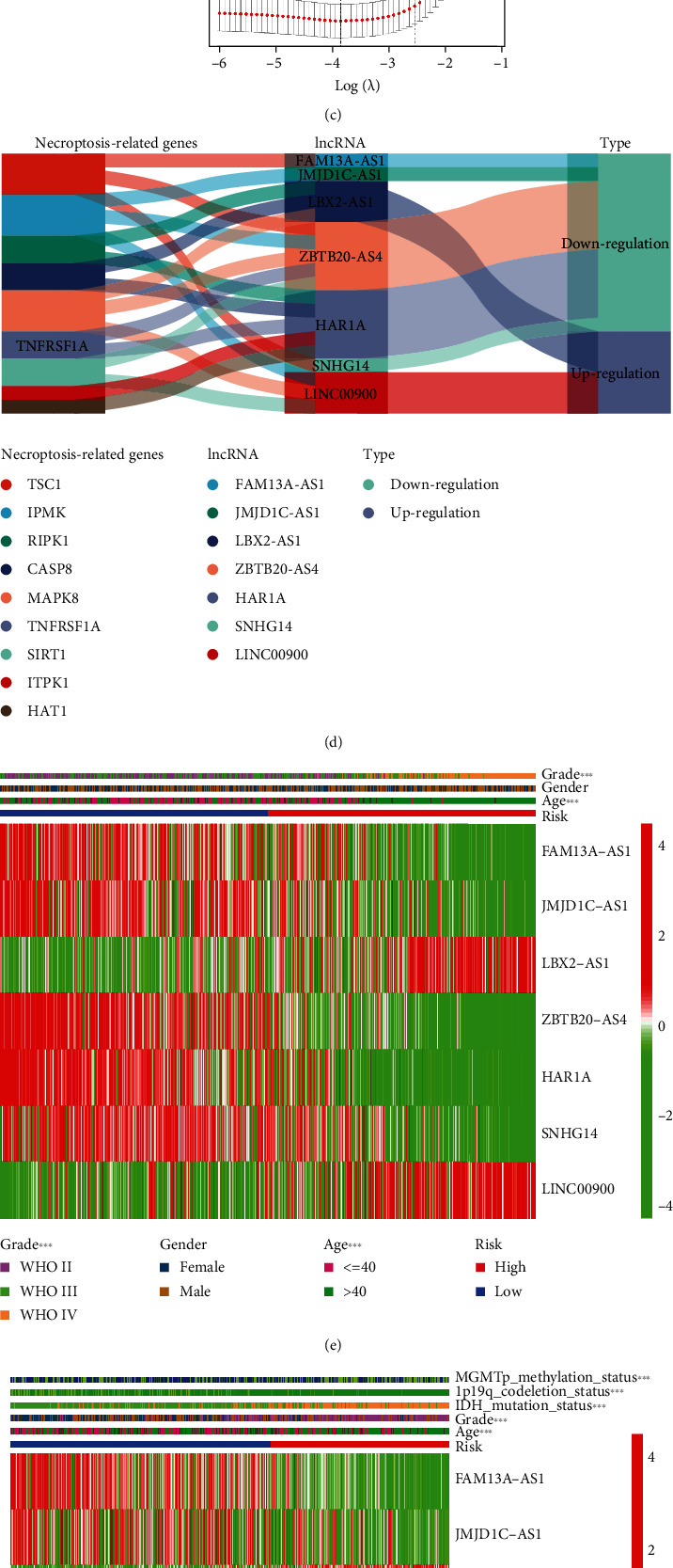
Establishment of a prognostic lncRNA model. (a) The lncRNAs with prognostic value extracted from the univariate regression analysis. (b, c) LASSO regression analysis was conducted to determine the final component lncRNAs of the prognostic model. (d) The Sankey diagram of model-related lncRNAs and associated necroptosis genes. (e, f) Heatmaps of the model-related lncRNAs and clinical features in the training cohort and validation cohort.

**Figure 3:**
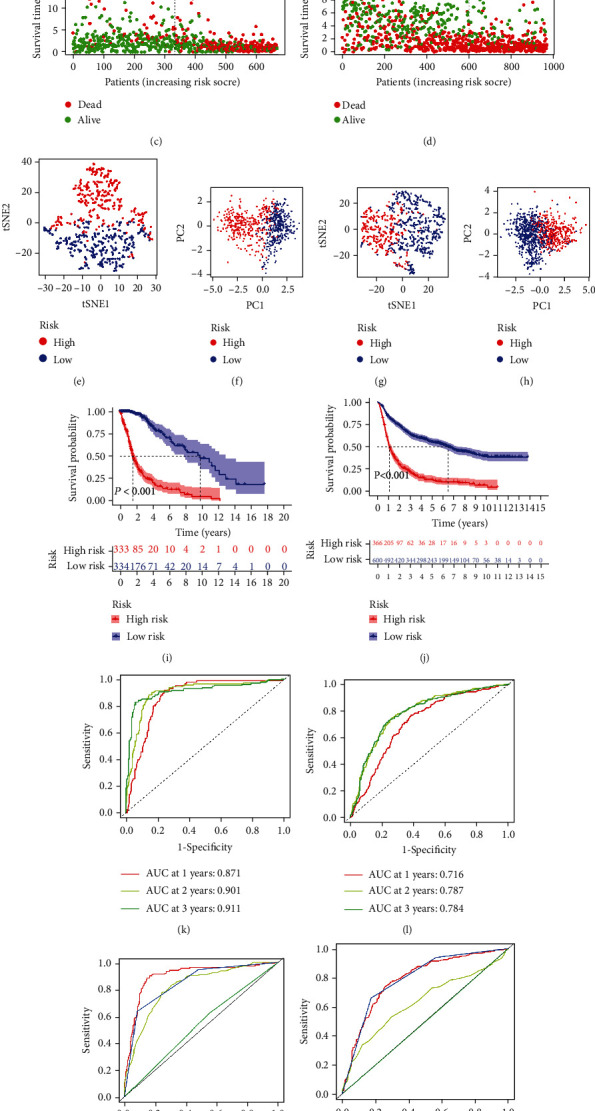
Validation of the prognostic model. (a, b) The median value and distribution of the risk scores in the training and validation cohorts. (c, d) The survival status of glioma patients with different risk scores in the training and validation cohorts. (e–h) The t-SNE and PCA plots of the training and validation cohorts. (i, j) The Kaplan-Meier curves of patients in the high-risk subgroup and low-risk subgroup in the training and validation cohorts. (k, l) The time-dependent ROC curves determined the prognostic efficacy of the risk score in the training and validation sets. (m, n) The 2-year ROC curves of risk score and clinical characteristics in the training and validation cohorts.

Based on the univariate regression analysis, we found that all 12 differentially expressed necroptosis-associated lncRNAs were significantly related to prognostic value (*p* < 0.001) (Figure 2(a)). We then did LASSO regression analysis on these lncRNAs and finally determined 7 lncRNAs to construct the prognostic model (Figures 2(b) and 2(c)). According to the model, we calculated the risk score for each glioma patient and classified the patients into two subgroups by median value. And we plotted heatmaps of the model-related lncRNAs and clinical characteristics between the two risk categories in the training cohort and the CGGA cohort, respectively (Figures 2(e) and 2(f)). The distributions of age, grade, IDH mutation status, 1p19q codeletion status, and MGMT methylation status were found to diverge between the low- and high-risk categories. MGMT methylation, IDH mutation, and 1p19q codeletion were more frequently seen in patients in the low-risk group compared to the high-risk group. And patients of advanced age and high pathological grade were more often clustered in the high-risk group. In addition, we also created a Sankey diagram (Figure 2(d)). The next step was to verify the effectiveness of the model. As shown in Figures 3(a) and 3(b), patients were separated into two subgroups based on the median cutoff value. And we found that patients in the high- and low-risk groups had dramatically different survival status (Figures 3(c) and 3(d)). The t-SNE and PCA analyses revealed that patients in both risk categories were distributed in different directions in the training cohort (Figures 3(e) and 3(f)) and validation cohort (Figures 3(g) and 3(h)). Depending on the Kaplan-Meier analysis, we discovered that patients in the low-risk subgroup had better results than those in the high-risk category (*p* < 0.001, Figures 3(i) and 3(j)). Finally, we performed ROC analysis in order to investigate the predictive power of this signature. The AUC of the lncRNA signature in the training set was 0.871, 0.901, and 0.911 at 1, 2, and 3 years, respectively (Figure 3(k)), while in the validation set the AUC was 0.716 at 1 year, 0.787 at 2 years, and 0.784 at 3 years (Figure 3(l)). And we observed superior predictive ability in our risk model compared to clinical characteristics (Figures 3(m) and 3(n)).

### 3.3. Independent Prognostic Value of the lncRNA Signature

**Figure 4:**
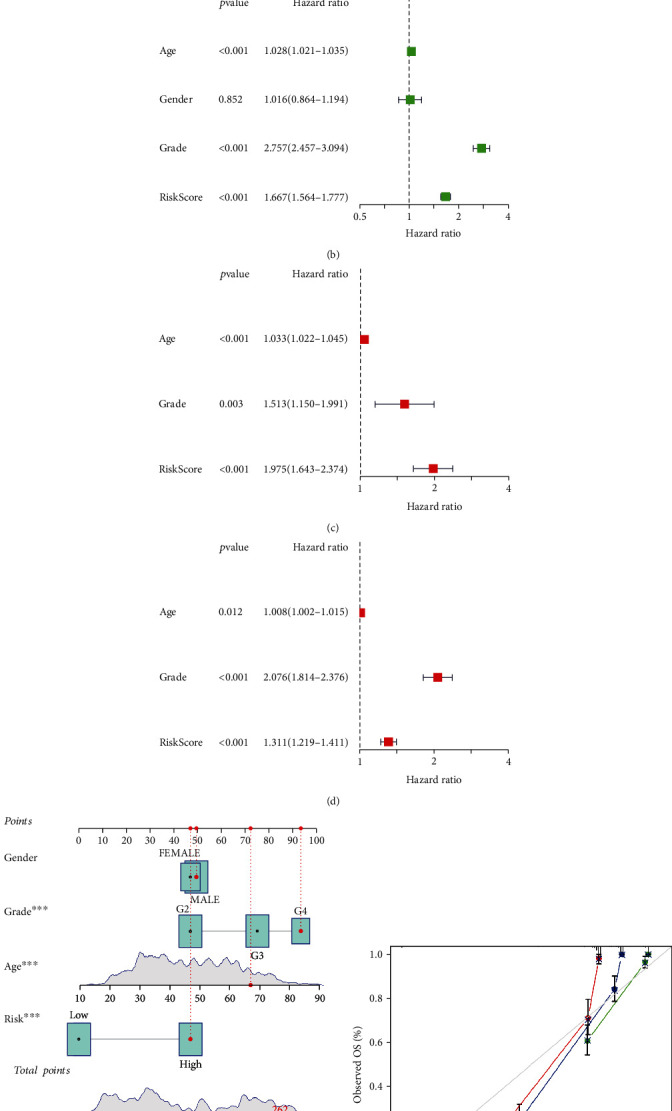
Nomogram and independent prognostic analysis. (a–d) The univariate and multivariate Cox regression analyses of the lncRNA signature and other clinical characteristics associated with OS in the training cohort and validation set. (e, f) The nomogram and calibration curves for predicting the probability of the 1-, 2-, and 3-year OS.

The univariate Cox regression analysis revealed that the risk scores in both the training and validation cohorts were significantly associated with overall survival (OS) (training set: HR: 3.002, 95% CI: 2.646-3.405, *p* < 0.001, Figure 4(a); validation set: HR: 1.667, 95% CI: 1.564-1.777, *p* < 0.001, Figure 4(b)). Furthermore, the multivariate Cox regression analysis indicated that the risk score could be considered an important independent prognostic predictor even when combined with other confounding factors (training set: HR: 1.975, 95% CI: 1.643-2.374, *p* < 0.001, Figure 4(c); validation set: HR: 1.311, 95% CI: 1.219-1.411, *p* < 0.001, Figure 4(d)). More details can be found in Table S5.

### 3.4. Creating and Verifying a Nomogram

Based on the Cox regression analysis, we constructed a nomogram to predict the prognosis of glioma patients (Figure 4(e)). The nomogram allowed us to score each patient and finally derive a total score to project the 1-, 2-, and 3-year OS occurrences. And as presented in Figure 4(f), we also plotted 1-, 2-, and 3-year calibration curves to assess the predictive value of the nomogram.

### 3.5. Functional Enrichment Analyses

**Figure 5:**
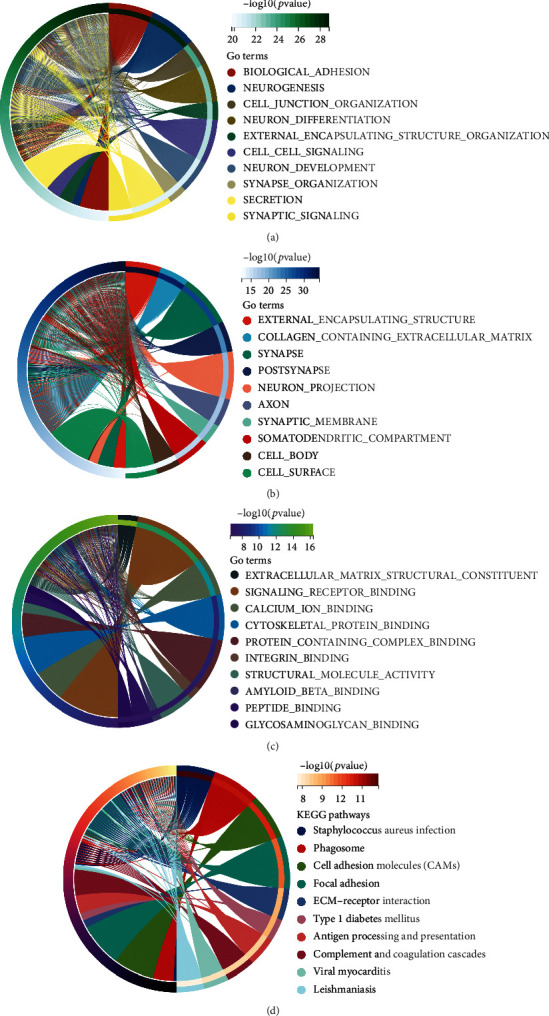
Functional enrichment analyses between the high-risk group and low-risk group. (a–c) The GO enrichment analysis between the two-risk subgroups. (d) The KEGG enrichment analysis between the two-risk subgroups.

The key biological activities of DEGs linked with the risk model were determined using GO and KEGG functional analyses. As shown in Figures 5(a)–5(c), the GO analysis suggested that the DEGs were significantly enriched in biological functions such as cell signaling and protein binding. Moreover, the KEGG pathway analysis indicated that the DEGs were remarkably concentrated in immune-related pathways, including phagosome, antigen processing, and presentation (*p* < 0.05, Figure 5(d)).

### 3.6. Exploration of the Immune Responses in Different Risk Subgroups

**Figure 6:**
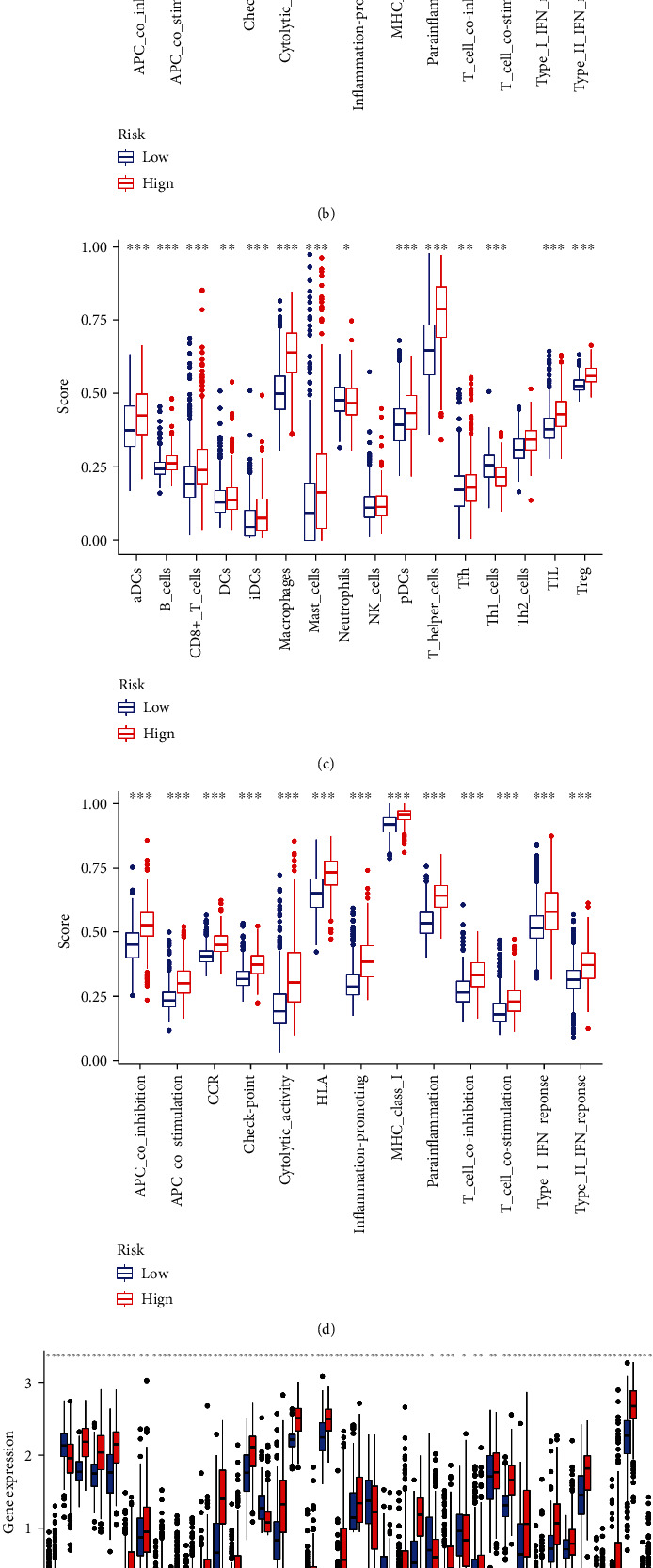
Immune-related analyses. (a, b) The boxplots showed the scores of 16 immune cells and 13 immune-related functions between the high-risk category and low-risk subgroup in the training cohort. (c, d) The results of the validation cohort. (e, f) The different expression levels of 32 immune checkpoints between the low-risk category and the high-risk category.

We performed the ssGSEA to investigate the immune responses of different risk categories. As presented in Figure 6(a), we found that most of the immune cells had higher infiltration levels in the high-risk group, except for the Th1 cells and neutrophils which infiltrated more in the low-risk category. Furthermore, in the high-risk subgroup, all 13 immune pathways were shown to be more active (Figure 6(b)). We observed comparable findings in the validation set (Figures 6(c) and 6(d)). As shown in Figures 6(e) and 6(f), we explored the differences in immune checkpoints expression between the high- and low-risk categories, and most immune checkpoints were more positive in the high risk class. All of these reflected that the immune response was more active in the high-risk group of glioma patients, and we could select appropriate therapeutic targets to improve the efficacy of immunotherapy.

### 3.7. Tumor Microenvironment Investigation

**Figure 7:**
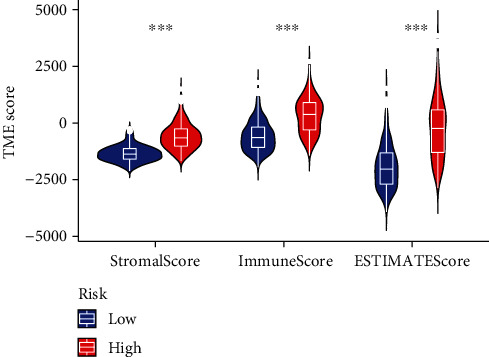
Investigation of the tumor microenvironment. Different stromal score and immune score between the high- and low-risk groups.

As presented in Figure 7, patients in the high-risk subgroup had higher stromal and immune scores. This may imply that the higher potential for patients in the high-risk group to benefit from immunotherapy. More details can be found in Table S6.

### 3.8. Validating the Expression Levels of Selected Necroptosis-Associated lncRNAs

**Figure 8:**
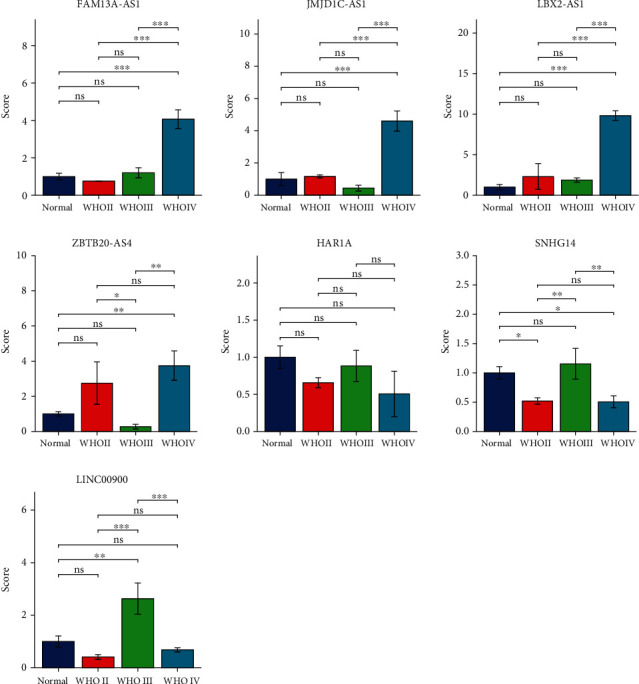
The qRT-PCR results of glioma tissues and matched normal tissues. The expression levels of selected necroptosis-related lncRNAs (FAM13A-AS1, JMJD1C-AS1, LBX2-AS1, ZBTB20-AS4, HAR1A, SNHG14, and LINC00900; ^∗^*p* < 0.05, ^∗∗^*p* < 0.01, and ^∗∗∗^*p* < 0.001).

As shown in Figure 8, we found that these selected necroptosis-associated lncRNAs were differentially expressed in tumor tissues and normal tissues, and the expression levels were also different between tumor tissues of different grades. These results further confirmed our previous conclusions.

## 4. Discussion

Immunotherapy first originated in the nineteenth century. In 1893, William Coley first treated cancer by activating the immune system. However, due to the immune evasion of tumor cells, which led to unsatisfactory treatment effect, the cancer immunotherapy did not attract much attention (22). In contrast, over the past few decades, immunotherapy has gradually become the first-line treatment option for many tumors as the tumor microenvironment and immune checkpoints have been studied in depth. Some studies have shown that PD-1/PD-L1 blockade has unprecedented efficacy in B cell lymphomas and can be used as an alternative to the failure of conventional treatment regimens (23, 24). According to Forde et al., PD-1 blockade is remarkably effective in the preoperative treatment of patients with early-stage lung cancer and has few side effects, which can significantly reduce the possibility of tumor recurrence (25). And some related studies found that CTLA-4 blockade can dramatically improve overall survival in melanoma (26, 27). Nevertheless, while the promising results of immunotherapy have been achieved, not all patients benefit from it, so we need to explore thoroughly which patients are suitable for immunotherapy in order to maximize the benefits of treatment.

Since most tumors are innately resistant to apoptosis, inducing new types of cell death has become a new strategy for cancer therapy. Necroptosis has gradually aroused attention as a form of proinflammatory programmed cell death. And recent research has shown that immunogenic substances released by necroptosis can exert powerful antitumor immune effects in concert with immune checkpoint blockade (28). Furthermore, a study by Yatim et al. found that necroptosis induces strong cross-priming of the immune system by coupling RIPK1 and NF-*κ*B to activate CD8(+) T cells (29). Therefore, combining necroptosis and immune checkpoint blockade seems to be a direction that holds great promise.

Traditional treatments for glioma include surgery, chemotherapy, and radiotherapy, but these do not significantly improve the survival of patients. The presence of the blood-brain barrier and the unique immune microenvironment of the brain make the treatment of glioma extremely difficult (30). Despite the remarkable results of many novel treatment options in other tumors, few drugs have been approved by the Food and Drug Administration (FDA) for glioma. However, a growing number of studies suggest that immunotherapy may become an important tool for the treatment of glioma in the future. A study by Wainwright et al. revealed that combined targeted inhibition of IDO, CTLA-4, and PD-L1 significantly prolonged the survival of glioma mice (31). In addition, Reardon et al. applied the combination of anti-CTLA-4 and anti-PD-1 therapy in glioma mouse models and cured 75% of the mice; moreover, these mice developed robust immune memory response (32).

In our study, we developed a prognostic signature based on necroptosis-associated lncRNAs. With this model, we assigned a unique risk score to each patient and classified the patients into the high- and low-risk groups. We found that the prognosis of patients in the high-risk group was significantly worse than that of the low-risk group. Validation analysis by ROC suggested that the model has strong predictive power and can assess the prognosis of glioma patients well. In addition, we created a nomogram to improve the clinical applicability of the model. By performing functional enrichment analyses of differential genes between the high- and low-risk categories, we found that these differential genes were mainly enriched in immune function-related pathways. Subsequently, we performed immune-related analysis, and most of the immune cells had higher infiltration levels in the high-risk group, such as CD8(+) T cells, macrophages, and Treg cells, suggesting the complexity of the immune microenvironment and potentially high response reactivity in high-risk group patients. The analysis of immune checkpoints revealed that the vast majority of immune checkpoints were highly expressed in patients of the high-risk group, especially the obvious targets like PD-1, IDO1, IDO2, and CTLA-4. And through the exploration of tumor microenvironment, we found that the immune score and stromal score of patients in the high-risk group were significantly higher than those in the low-risk group. All these implied that modulating the immune microenvironment of patients by targeting immune checkpoints has great potential value in the high-risk group patients.

Our model included 7 lncRNAs (FAM13A-AS1, JMJD1C-AS1, LBX2-AS1, ZBTB20-AS4, HAR1A, SNHG14, and LINC00900). Among them, LBX2-AS1 was found to be closely associated with the progression of several cancers. According to Yang et al., LBX2-AS1 is highly expressed in gastric cancer tissues and mainly affects the proliferation of gastric cancer cells (33). According to Cao et al., LBX2-AS1 is considered to be an oncogenic lncRNA in ovarian cancer, and targeted knockdown of LBX2-AS1 significantly reduces the ability of ovarian cancer cells to grow and invade (34). In addition, it was found that high expression of LBX2-AS1 in glioma patients was associated with poor prognosis (35). HAR1A is a tumor suppressor, and in oral cancer patients, knockdown of HAR1A promotes the expression of ALPK1 and leads to oral cancer progression (36). According to Dong et al., SNHG14 plays a critical role in the mechanism of drug resistance in breast cancer, and knockdown of SNHG14 significantly improves trastuzumab efficacy in breast cancer patients (37). According to Zhao et al., SNHG14 is highly expressed in diffuse large B cell lymphoma, and the SNHG14/miR-5590-3p/ZEB1 pathway would enable tumor immune evasion by regulating the immune checkpoint PD-1; targeted inhibition of SNHG14 expression is likely to improve the efficacy of immunotherapy (38).

At the same time, there are some limitations in this work. First, most of the data we analyzed were obtained from publicly available databases, so it is necessary for us to conduct more profound in vivo and in vitro trials to validate our results. Second, some lncRNAs in our model have not been studied in depth and await further exploration. This motivates us to keep exploring further in order to contribute to the treatment of glioma.

## 5. Conclusions

In conclusion, the necroptosis-associated lncRNA signature we created can accurately predict patient's prognosis, and the combination of necroptosis-related lncRNAs and immune checkpoints blockade has the potential to be a promising new therapeutic option. Our study provides new ideas for individualized treatment of glioma patients.

## Data Availability

TCGA (https://portal.gdc.cancer.gov), CGGA (http://www.cgga.org.cn/), and GTEx (https://gtexportal.org/home/datasets) belong to public datasets. Our research is based on these publicly available datasets. The original contributions presented in the study are included in the article/Supplementary Material; further inquiries can be directed to the corresponding author.

## Supplementary Materials

Supplementary 1 Table S1: 67 necroptosis-associated genes.

Supplementary 2 Table S2: the cor-expression results of necroptosis-related genes and lncRNAs.

Supplementary 3 Table S3: 12 differentially expressed necroptosis-associated lncRNAs.

Supplementary 4 Table S4: the uniCox results of the necroptosis-associated lncRNAs.

Supplementary 5 Table S5: independent prognostic analyses of the training and testing sets.

Supplementary 6 Table S6: the investigation of tumor microenvironment of the training cohort.
