# Supplementary material for: A Novel Necroptosis-Related lncRNA Signature for Predicting Prognosis and Immune Response of Glioma
Source: Biomed Res Int. 2022 Jun 16;2022:3742447. doi: 10.1155/2022/3742447 (PMC9226973; doi:10.1155/2022/3742447)
Supplement: Supplementary 1 — Table S1: 67 necroptosis-associated genes. [file 3742447.f1.docx]

Table S1 67 necroptosis-associated genes.

| Gene_name |
| --- |
| FADD |
| FAS |
| FASLG |
| MLKL |
| RIPK1 |
| RIPK3 |
| TLR3 |
| TNF |
| TSC1 |
| TRIM11 |
| CASP8 |
| ZBP1 |
| MAPK8 |
| IPMK |
| ITPK1 |
| SIRT3 |
| MYC |
| TNFRSF1A |
| TNFSF10 |
| TNFRSF1B |
| TRAF2 |
| PANX1 |
| OTULIN |
| CYLD |
| USP22 |
| MAP3K7 |
| SQSTM1 |
| STAT3 |
| DIABLO |
| DNMT1 |
| CFLAR |
| BRAF |
| AXL |
| ID1 |
| CDKN2A |
| HSPA4 |
| BCL2 |
| STUB1 |
| FLT3 |
| HAT1 |
| SIRT2 |
| SIRT1 |
| PLK1 |
| MPG |
| BACH2 |
| GATA3 |
| MYCN |
| ALK |
| ATRX |
| TERT |
| SLC39A7 |
| SPATA2 |
| RNF31 |
| IDH1 |
| IDH2 |
| KLF9 |
| HDAC9 |
| HSP90AA1 |
| LEF1 |
| BNIP3 |
| CD40 |
| BCL2L11 |
| EGFR |
| DDX58 |
| TARDBP |
| APP |
| TNFRSF21 |
