# Supplementary material for: A Novel Necroptosis-Related lncRNA Signature for Predicting Prognosis and Immune Response of Glioma
Source: Biomed Res Int. 2022 Jun 16;2022:3742447. doi: 10.1155/2022/3742447 (PMC9226973; doi:10.1155/2022/3742447)
Supplement: Supplementary 2 — Table S2: the cor-expression results of necroptosis-related genes and lncRNAs. [file 3742447.f2.docx]

Table S2 The cor-expression results of necroptosis-related genes and lncRNAs

| NecrGene | lncRNA | cor | pvalue | Regulation |
| --- | --- | --- | --- | --- |
| MYCN | MYCNOS | 0.864563377 | 3.54E-210 | postive |
| ITPK1 | STXBP5-AS1 | 0.608448732 | 6.54E-72 | postive |
| IPMK | FAM181A-AS1 | -0.602178653 | 4.27E-70 | negative |
| TNFRSF1A | FAM181A-AS1 | 0.615268743 | 6.23E-74 | postive |
| MLKL | USP30-AS1 | 0.638158207 | 4.32E-81 | postive |
| RIPK3 | USP30-AS1 | 0.651229938 | 1.85E-85 | postive |
| CASP8 | USP30-AS1 | 0.66744748 | 3.44E-91 | postive |
| TNFRSF1A | USP30-AS1 | 0.644692389 | 3.01E-83 | postive |
| CD40 | USP30-AS1 | 0.627442736 | 1.15E-77 | postive |
| PLK1 | H19 | 0.620010329 | 2.29E-75 | postive |
| TSC1 | HOTAIRM1 | -0.626620883 | 2.08E-77 | negative |
| MAPK8 | HOTAIRM1 | -0.685664395 | 4.50E-98 | negative |
| IPMK | HOTAIRM1 | -0.637813156 | 5.59E-81 | negative |
| SIRT1 | HOTAIRM1 | -0.675042221 | 5.34E-94 | negative |
| RIPK1 | LINC00632 | -0.655601932 | 5.71E-87 | negative |
| TSC1 | LINC00632 | 0.695744625 | 4.19E-102 | postive |
| CASP8 | LINC00632 | -0.672400841 | 5.17E-93 | negative |
| MAPK8 | LINC00632 | 0.709873519 | 4.79E-108 | postive |
| IPMK | LINC00632 | 0.601823319 | 5.40E-70 | postive |
| TNFRSF1A | LINC00632 | -0.686579638 | 1.97E-98 | negative |
| TSC1 | TMEM72-AS1 | 0.680792226 | 3.49E-96 | postive |
| MAPK8 | TMEM72-AS1 | 0.673456207 | 2.09E-93 | postive |
| IPMK | TMEM72-AS1 | 0.604462752 | 9.42E-71 | postive |
| SIRT1 | TMEM72-AS1 | 0.63420682 | 8.21E-80 | postive |
| IPMK | RGMB-AS1 | 0.676081346 | 2.17E-94 | postive |
| SIRT1 | RGMB-AS1 | 0.667081754 | 4.68E-91 | postive |
| LEF1 | LEF1-AS1 | 0.704215405 | 1.27E-105 | postive |
| MAPK8 | STX18-AS1 | 0.606842034 | 1.92E-71 | postive |
| SIRT1 | STX18-AS1 | 0.644043402 | 4.95E-83 | postive |
| PLK1 | CASC2 | -0.626818249 | 1.81E-77 | negative |
| USP22 | ZSCAN16-AS1 | -0.613700685 | 1.83E-73 | negative |
| TSC1 | GDNF-AS1 | 0.669045426 | 8.97E-92 | postive |
| CASP8 | GDNF-AS1 | -0.669473639 | 6.24E-92 | negative |
| MAPK8 | GDNF-AS1 | 0.706826979 | 9.81E-107 | postive |
| IPMK | GDNF-AS1 | 0.732955269 | 1.46E-118 | postive |
| TNFRSF1A | GDNF-AS1 | -0.700813953 | 3.39E-104 | negative |
| SIRT1 | GDNF-AS1 | 0.725151298 | 6.92E-115 | postive |
| TSC1 | LINC00339 | -0.605581064 | 4.47E-71 | negative |
| ITPK1 | LINC00339 | -0.627231796 | 1.34E-77 | negative |
| FADD | LINC00641 | -0.725809768 | 3.43E-115 | negative |
| RIPK1 | LINC00641 | -0.711355461 | 1.09E-108 | negative |
| TSC1 | LINC00641 | 0.729959195 | 3.90E-117 | postive |
| CASP8 | LINC00641 | -0.691580249 | 2.03E-100 | negative |
| MAPK8 | LINC00641 | 0.759344961 | 5.10E-132 | postive |
| ITPK1 | LINC00641 | 0.679517492 | 1.08E-95 | postive |
| TNFRSF1A | LINC00641 | -0.665594983 | 1.62E-90 | negative |
| BRAF | LINC00641 | 0.674572511 | 8.00E-94 | postive |
| HAT1 | LINC00641 | -0.651601256 | 1.38E-85 | negative |
| SIRT1 | LINC00641 | 0.657073028 | 1.75E-87 | postive |
| PLK1 | LINC00641 | -0.601386953 | 7.19E-70 | negative |
| MLKL | PCED1B-AS1 | 0.627072399 | 1.50E-77 | postive |
| RIPK3 | PCED1B-AS1 | 0.844701757 | 4.19E-191 | postive |
| TLR3 | PCED1B-AS1 | 0.649124707 | 9.65E-85 | postive |
| CASP8 | PCED1B-AS1 | 0.681521882 | 1.83E-96 | postive |
| TNFRSF1A | PCED1B-AS1 | 0.704285659 | 1.18E-105 | postive |
| TNFRSF1B | PCED1B-AS1 | 0.78896471 | 2.31E-149 | postive |
| BRAF | PCED1B-AS1 | -0.632312424 | 3.32E-79 | negative |
| CD40 | PCED1B-AS1 | 0.755825302 | 3.99E-130 | postive |
| FADD | MIR600HG | -0.633176361 | 1.76E-79 | negative |
| RIPK1 | MIR600HG | -0.603390475 | 1.92E-70 | negative |
| TSC1 | MIR600HG | 0.705825252 | 2.62E-106 | postive |
| CASP8 | MIR600HG | -0.633088351 | 1.87E-79 | negative |
| TNFRSF1A | MIR600HG | -0.662701191 | 1.78E-89 | negative |
| USP22 | MIR600HG | 0.62791277 | 8.20E-78 | postive |
| BRAF | MIR600HG | 0.667397708 | 3.59E-91 | postive |
| HAT1 | MIR600HG | -0.659715231 | 2.06E-88 | negative |
| RIPK1 | EPB41L4A-AS1 | -0.602067496 | 4.60E-70 | negative |
| CASP8 | EPB41L4A-AS1 | -0.686447022 | 2.22E-98 | negative |
| MAPK8 | EPB41L4A-AS1 | 0.629833101 | 2.03E-78 | postive |
| TNFRSF1A | EPB41L4A-AS1 | -0.672340423 | 5.45E-93 | negative |
| SIRT1 | EPB41L4A-AS1 | 0.631788887 | 4.87E-79 | postive |
| CASP8 | ARHGEF7-AS2 | -0.658509523 | 5.48E-88 | negative |
| MAPK8 | ARHGEF7-AS2 | 0.611846318 | 6.53E-73 | postive |
| TNFRSF1A | ARHGEF7-AS2 | -0.64536536 | 1.79E-83 | negative |
| MAPK8 | CRNDE | -0.613058974 | 2.85E-73 | negative |
| ITPK1 | FAM201A | 0.6065669 | 2.31E-71 | postive |
| PLK1 | LINC00882 | -0.630920367 | 9.20E-79 | negative |
| TSC1 | ZNF32-AS2 | 0.68370755 | 2.61E-97 | postive |
| SIRT1 | ZNF32-AS2 | 0.627412585 | 1.18E-77 | postive |
| MAPK8 | ACVR2B-AS1 | 0.617145293 | 1.70E-74 | postive |
| IPMK | ACVR2B-AS1 | 0.613196242 | 2.59E-73 | postive |
| SIRT1 | ACVR2B-AS1 | 0.647724406 | 2.88E-84 | postive |
| RIPK1 | PAXBP1-AS1 | -0.615796367 | 4.32E-74 | negative |
| TSC1 | PAXBP1-AS1 | 0.697981382 | 5.06E-103 | postive |
| CASP8 | PAXBP1-AS1 | -0.676565399 | 1.42E-94 | negative |
| MAPK8 | PAXBP1-AS1 | 0.688792284 | 2.63E-99 | postive |
| IPMK | PAXBP1-AS1 | 0.624034872 | 1.33E-76 | postive |
| ITPK1 | PAXBP1-AS1 | 0.625735776 | 3.93E-77 | postive |
| TNFRSF1A | PAXBP1-AS1 | -0.734051957 | 4.34E-119 | negative |
| SIRT1 | PAXBP1-AS1 | 0.683536497 | 3.04E-97 | postive |
| CD40 | PAXBP1-AS1 | -0.629050677 | 3.59E-78 | negative |
| ITPK1 | HHATL-AS1 | 0.639986167 | 1.09E-81 | postive |
| FADD | OIP5-AS1 | -0.707752166 | 3.94E-107 | negative |
| BRAF | OIP5-AS1 | 0.658865167 | 4.11E-88 | postive |
| FAS | MIR155HG | 0.627011616 | 1.57E-77 | postive |
| RIPK1 | MIR155HG | 0.639438345 | 1.65E-81 | postive |
| TSC1 | MIR155HG | -0.684052971 | 1.92E-97 | negative |
| CASP8 | MIR155HG | 0.673153427 | 2.71E-93 | postive |
| MAPK8 | MIR155HG | -0.729421269 | 6.99E-117 | negative |
| IPMK | MIR155HG | -0.626271729 | 2.68E-77 | negative |
| ITPK1 | MIR155HG | -0.601926387 | 5.04E-70 | negative |
| TNFRSF1A | MIR155HG | 0.66713715 | 4.47E-91 | postive |
| SIRT1 | MIR155HG | -0.661418653 | 5.11E-89 | negative |
| SIRT3 | BDNF-AS | 0.615324818 | 5.99E-74 | postive |
| CDKN2A | CDKN2B-AS1 | 0.804079138 | 2.49E-159 | postive |
| IPMK | FAM222A-AS1 | 0.640315781 | 8.48E-82 | postive |
| SIRT1 | FAM222A-AS1 | 0.681779518 | 1.46E-96 | postive |
| TSC1 | MORF4L2-AS1 | 0.639233593 | 1.92E-81 | postive |
| TNFRSF1A | MORF4L2-AS1 | -0.620064802 | 2.20E-75 | negative |
| SIRT1 | MORF4L2-AS1 | 0.60220649 | 4.19E-70 | postive |
| SIRT1 | NNT-AS1 | 0.603499696 | 1.78E-70 | postive |
| TSC1 | DNAH10OS | 0.600071402 | 1.70E-69 | postive |
| BRAF | DNAH10OS | 0.612327302 | 4.70E-73 | postive |
| TSC1 | FAM13A-AS1 | 0.653816187 | 2.38E-86 | postive |
| TSC1 | ZNF571-AS1 | 0.616724485 | 2.27E-74 | postive |
| RIPK1 | SLC25A21-AS1 | -0.647722705 | 2.88E-84 | negative |
| TSC1 | SLC25A21-AS1 | 0.665610483 | 1.60E-90 | postive |
| CASP8 | SLC25A21-AS1 | -0.661810157 | 3.71E-89 | negative |
| MAPK8 | SLC25A21-AS1 | 0.725254041 | 6.20E-115 | postive |
| IPMK | SLC25A21-AS1 | 0.728453915 | 2.00E-116 | postive |
| ITPK1 | SLC25A21-AS1 | 0.623040172 | 2.69E-76 | postive |
| TNFRSF1A | SLC25A21-AS1 | -0.650656735 | 2.90E-85 | negative |
| SIRT1 | SLC25A21-AS1 | 0.766231965 | 8.04E-136 | postive |
| RIPK1 | BCDIN3D-AS1 | -0.649972805 | 4.97E-85 | negative |
| MAPK8 | BCDIN3D-AS1 | 0.663283054 | 1.10E-89 | postive |
| TNFRSF1A | BCDIN3D-AS1 | -0.653444722 | 3.20E-86 | negative |
| BRAF | BCDIN3D-AS1 | 0.608023347 | 8.71E-72 | postive |
| TSC1 | SNHG18 | -0.620131541 | 2.10E-75 | negative |
| MAPK8 | SNHG18 | -0.690422226 | 5.90E-100 | negative |
| IPMK | SNHG18 | -0.651175974 | 1.93E-85 | negative |
| SIRT1 | SNHG18 | -0.651469432 | 1.53E-85 | negative |
| IPMK | LINC00092 | -0.681757329 | 1.48E-96 | negative |
| TSC1 | AGAP2-AS1 | -0.616610505 | 2.46E-74 | negative |
| MAPK8 | AGAP2-AS1 | -0.631185607 | 7.58E-79 | negative |
| IPMK | AGAP2-AS1 | -0.63353867 | 1.34E-79 | negative |
| SIRT1 | AGAP2-AS1 | -0.679364162 | 1.23E-95 | negative |
| TSC1 | TRAPPC12-AS1 | 0.673160997 | 2.70E-93 | postive |
| MAPK8 | TRAPPC12-AS1 | 0.621721537 | 6.86E-76 | postive |
| IPMK | JMJD1C-AS1 | 0.619951642 | 2.39E-75 | postive |
| IPMK | LINC00630 | 0.648102174 | 2.15E-84 | postive |
| BRAF | LINC00630 | 0.610298924 | 1.87E-72 | postive |
| SIRT1 | LINC00630 | 0.642630557 | 1.46E-82 | postive |
| TSC1 | SBF2-AS1 | -0.600358025 | 1.41E-69 | negative |
| IPMK | SBF2-AS1 | -0.624731923 | 8.07E-77 | negative |
| SIRT1 | SBF2-AS1 | -0.634588789 | 6.18E-80 | negative |
| RIPK1 | LBX2-AS1 | 0.617095408 | 1.76E-74 | postive |
| CASP8 | LBX2-AS1 | 0.630493385 | 1.26E-78 | postive |
| MAPK8 | LBX2-AS1 | -0.646155114 | 9.73E-84 | negative |
| RIPK1 | WAC-AS1 | -0.632516162 | 2.86E-79 | negative |
| TSC1 | WAC-AS1 | 0.638369533 | 3.68E-81 | postive |
| CASP8 | WAC-AS1 | -0.614877272 | 8.16E-74 | negative |
| MAPK8 | WAC-AS1 | 0.695724946 | 4.26E-102 | postive |
| IPMK | WAC-AS1 | 0.615448025 | 5.50E-74 | postive |
| TNFRSF1A | WAC-AS1 | -0.637794501 | 5.67E-81 | negative |
| SIRT1 | WAC-AS1 | 0.628764291 | 4.42E-78 | postive |
| TSC1 | LINC00928 | 0.602142174 | 4.37E-70 | postive |
| MAPK8 | LINC00928 | 0.621725873 | 6.84E-76 | postive |
| IPMK | LINC00928 | 0.632602753 | 2.68E-79 | postive |
| SIRT1 | LINC00928 | 0.631141496 | 7.83E-79 | postive |
| TSC1 | NCAM1-AS1 | 0.651325438 | 1.71E-85 | postive |
| RIPK1 | HCP5 | 0.639996293 | 1.08E-81 | postive |
| TSC1 | HCP5 | -0.628831765 | 4.21E-78 | negative |
| CASP8 | HCP5 | 0.696734796 | 1.65E-102 | postive |
| MAPK8 | HCP5 | -0.62579173 | 3.78E-77 | negative |
| TNFRSF1A | HCP5 | 0.646853294 | 5.66E-84 | postive |
| TNFSF10 | HCP5 | 0.6430094 | 1.09E-82 | postive |
| CASP8 | SNAI3-AS1 | -0.608225534 | 7.60E-72 | negative |
| ITPK1 | SNAI3-AS1 | 0.630352972 | 1.39E-78 | postive |
| STAT3 | SNAI3-AS1 | -0.609149318 | 4.07E-72 | negative |
| STUB1 | SNAI3-AS1 | 0.631178472 | 7.62E-79 | postive |
| TSC1 | ZBTB20-AS4 | 0.609931771 | 2.40E-72 | postive |
| MAPK8 | ZBTB20-AS4 | 0.692823885 | 6.41E-101 | postive |
| IPMK | ZBTB20-AS4 | 0.711803474 | 6.94E-109 | postive |
| TNFRSF1A | ZBTB20-AS4 | -0.602574947 | 3.29E-70 | negative |
| SIRT1 | ZBTB20-AS4 | 0.776454234 | 1.03E-141 | postive |
| IPMK | LINC00237 | 0.601255941 | 7.84E-70 | postive |
| RIPK1 | PAXIP1-AS2 | 0.601250925 | 7.86E-70 | postive |
| TRIM11 | PAXIP1-AS2 | -0.603247435 | 2.11E-70 | negative |
| CASP8 | PAXIP1-AS2 | 0.665109954 | 2.43E-90 | postive |
| MAPK8 | PAXIP1-AS2 | -0.638789775 | 2.68E-81 | negative |
| TNFRSF1A | PAXIP1-AS2 | 0.606676721 | 2.15E-71 | postive |
| BRAF | RAB11B-AS1 | -0.625113486 | 6.14E-77 | negative |
| MAPK8 | LINC00863 | 0.653050387 | 4.37E-86 | postive |
| IPMK | LINC00863 | 0.689856096 | 9.93E-100 | postive |
| SIRT1 | LINC00863 | 0.68668983 | 1.78E-98 | postive |
| TSC1 | C1orf220 | 0.6743659 | 9.57E-94 | postive |
| MAPK8 | C1orf220 | 0.636290168 | 1.75E-80 | postive |
| IPMK | C1orf220 | 0.625768472 | 3.84E-77 | postive |
| SIRT1 | C1orf220 | 0.648936193 | 1.12E-84 | postive |
| RIPK1 | HAR1A | -0.617248026 | 1.58E-74 | negative |
| CASP8 | HAR1A | -0.650281984 | 3.90E-85 | negative |
| ITPK1 | HAR1A | 0.605623967 | 4.35E-71 | postive |
| TNFRSF1A | HAR1A | -0.654710963 | 1.17E-86 | negative |
| HAT1 | HAR1A | -0.618920079 | 4.92E-75 | negative |
| TNFRSF1A | HAR1B | -0.601076563 | 8.82E-70 | negative |
| TSC1 | SNHG14 | 0.65229579 | 7.96E-86 | postive |
| RIPK1 | DGCR5 | -0.670573823 | 2.46E-92 | negative |
| CASP8 | DGCR5 | -0.708437503 | 2.00E-107 | negative |
| MAPK8 | DGCR5 | 0.667553399 | 3.15E-91 | postive |
| ITPK1 | DGCR5 | 0.604765012 | 7.70E-71 | postive |
| TNFRSF1A | DGCR5 | -0.659763007 | 1.98E-88 | negative |
| HAT1 | DGCR5 | -0.636292723 | 1.74E-80 | negative |
| ITPK1 | LINC00320 | 0.648117769 | 2.12E-84 | postive |
| RIPK1 | TOB1-AS1 | -0.657827614 | 9.52E-88 | negative |
| CASP8 | TOB1-AS1 | -0.682832594 | 5.70E-97 | negative |
| TNFRSF1A | TOB1-AS1 | -0.658698358 | 4.70E-88 | negative |
| FAS | LINC00601 | 0.613597686 | 1.97E-73 | postive |
| CASP8 | LINC00601 | 0.620979514 | 1.16E-75 | postive |
| MAPK8 | LINC00601 | -0.638660979 | 2.96E-81 | negative |
| TNFRSF1A | LINC00601 | 0.608095815 | 8.29E-72 | postive |
| RIPK3 | ITGB2-AS1 | 0.644045704 | 4.94E-83 | postive |
| MAPK8 | LINC00900 | -0.614971503 | 7.65E-74 | negative |
| IPMK | LINC00900 | -0.628927771 | 3.93E-78 | negative |
| SIRT1 | LINC00900 | -0.6147426 | 8.95E-74 | negative |
| PLK1 | WDFY3-AS2 | -0.672887695 | 3.41E-93 | negative |
| TSC1 | PVT1 | -0.655910941 | 4.46E-87 | negative |
| MAPK8 | PVT1 | -0.64301062 | 1.09E-82 | negative |
| ITPK1 | PVT1 | -0.638480104 | 3.39E-81 | negative |
| SIRT1 | PVT1 | -0.624256267 | 1.13E-76 | negative |
| MAPK8 | TMEM254-AS1 | 0.600471073 | 1.31E-69 | postive |
| TSC1 | MCM3AP-AS1 | 0.621079866 | 1.08E-75 | postive |
| FADD | ACAP2-IT1 | -0.65015528 | 4.30E-85 | negative |
| BRAF | ACAP2-IT1 | 0.701972411 | 1.11E-104 | postive |
