# Supplementary material for: A Novel Necroptosis-Related lncRNA Signature for Predicting Prognosis and Immune Response of Glioma
Source: Biomed Res Int. 2022 Jun 16;2022:3742447. doi: 10.1155/2022/3742447 (PMC9226973; doi:10.1155/2022/3742447)
Supplement: Supplementary 3 — Table S3: 12 differentially expressed necroptosis-associated lncRNAs. [file 3742447.f3.docx]

Table S3 12 differentially expressed necroptosis-associated lncRNAs.

| gene | conMean | treatMean | logFC | pValue | fdr |
| --- | --- | --- | --- | --- | --- |
| STXBP5-AS1 | 1.236050117 | 0.397166715 | -1.637920613 | 2.46E-213 | 2.00E-212 |
| USP30-AS1 | 0.27993081 | 0.586957425 | 1.068185575 | 7.64E-47 | 1.34E-46 |
| LINC00632 | 2.800831877 | 1.087959563 | -1.364230451 | 3.07E-270 | 9.98E-269 |
| FAM13A-AS1 | 1.557696626 | 0.714121586 | -1.125172651 | 1.88E-237 | 2.45E-236 |
| JMJD1C-AS1 | 0.569786839 | 1.529059188 | 1.424150048 | 2.00E-184 | 1.08E-183 |
| LBX2-AS1 | 0.484381534 | 1.131946876 | 1.224590479 | 5.04E-175 | 2.52E-174 |
| LINC00928 | 0.251691721 | 1.274786104 | 2.340525529 | 1.66E-209 | 1.20E-208 |
| ZBTB20-AS4 | 0.273347731 | 0.818414647 | 1.582094566 | 7.13E-117 | 2.01E-116 |
| LINC00237 | 1.102695522 | 2.423036685 | 1.13578176 | 1.23E-146 | 5.31E-146 |
| HAR1A | 1.665015526 | 0.788139164 | -1.079013332 | 8.32E-118 | 2.46E-117 |
| SNHG14 | 4.820032617 | 2.306813848 | -1.063141321 | 2.25E-285 | 1.46E-283 |
| LINC00900 | 1.254281546 | 0.620709698 | -1.014870632 | 8.46E-127 | 2.75E-126 |
