# Supplementary material for: A Novel Necroptosis-Related lncRNA Signature for Predicting Prognosis and Immune Response of Glioma
Source: Biomed Res Int. 2022 Jun 16;2022:3742447. doi: 10.1155/2022/3742447 (PMC9226973; doi:10.1155/2022/3742447)
Supplement: Supplementary 4 — Table S4: the uniCox results of the necroptosis-associated lncRNAs. [file 3742447.f4.docx]

Table S4 The uniCox results of the necroptosis-associated lncRNAs.

| gene | HR | HR.95L | HR.95H | pvalue |
| --- | --- | --- | --- | --- |
| STXBP5-AS1 | 0.118270297 | 0.06759176 | 0.206946278 | 7.52E-14 |
| USP30-AS1 | 2.514149596 | 2.129803052 | 2.967855731 | 1.27E-27 |
| LINC00632 | 0.210749619 | 0.166925278 | 0.266079545 | 3.71E-39 |
| FAM13A-AS1 | 0.045380937 | 0.026217039 | 0.078553093 | 2.26E-28 |
| JMJD1C-AS1 | 0.311268184 | 0.254710961 | 0.380383639 | 3.84E-30 |
| LBX2-AS1 | 3.603612773 | 2.894691481 | 4.486151667 | 1.87E-30 |
| LINC00928 | 0.317728161 | 0.258192709 | 0.390991616 | 2.47E-27 |
| ZBTB20-AS4 | 0.115866234 | 0.085177403 | 0.15761204 | 6.83E-43 |
| LINC00237 | 0.521254783 | 0.457994911 | 0.593252331 | 5.63E-23 |
| HAR1A | 0.225661926 | 0.173521854 | 0.293469115 | 1.18E-28 |
| SNHG14 | 0.176781217 | 0.135628468 | 0.230420643 | 1.32E-37 |
| LINC00900 | 4.756627112 | 3.689663053 | 6.132132164 | 2.36E-33 |
