# Supplementary material for: A Novel Necroptosis-Related lncRNA Signature for Predicting Prognosis and Immune Response of Glioma
Source: Biomed Res Int. 2022 Jun 16;2022:3742447. doi: 10.1155/2022/3742447 (PMC9226973; doi:10.1155/2022/3742447)
Supplement: Supplementary 5 — Table S5: independent prognostic analyses of the training and testing sets. [file 3742447.f5.docx]

Table S5 Independent prognostic analyses of the training and testing sets

| Training set |  |  |  |  |
| --- | --- | --- | --- | --- |
| uniCox |  |  |  |  |
| id | HR | HR.95L | HR.95H | pvalue |
| Age | 1.067003862 | 1.056997628 | 1.07710482 | 1.77E-41 |
| Gender | 1.233635174 | 0.957622717 | 1.589201796 | 0.104191155 |
| Grade | 4.665499147 | 3.834941743 | 5.675935581 | 1.68E-53 |
| riskScore | 3.001911056 | 2.646373866 | 3.405214245 | 1.73E-65 |
|  |  |  |  |  |
| multiCox |  |  |  |  |
| id | HR | HR.95L | HR.95H | pvalue |
| Age | 1.033331056 | 1.022208625 | 1.044574507 | 2.88E-09 |
| Grade | 1.513398259 | 1.150425072 | 1.990893928 | 0.003061251 |
| riskScore | 1.974971287 | 1.642680458 | 2.374479812 | 4.47E-13 |

| Testing set |  |  |  |  |
| --- | --- | --- | --- | --- |
| uniCox |  |  |  |  |
| id | HR | HR.95L | HR.95H | pvalue |
| Age | 1.028180947 | 1.021051728 | 1.035359943 | 4.94E-15 |
| Gender | 1.015549393 | 0.863756591 | 1.194017598 | 0.851817769 |
| Grade | 2.757139456 | 2.457331946 | 3.09352507 | 8.28E-67 |
| riskScore | 1.667050264 | 1.563904975 | 1.776998364 | 1.99E-55 |
|  |  |  |  |  |
| multiCox |  |  |  |  |
| id | HR | HR.95L | HR.95H | pvalue |
| Age | 1.008326302 | 1.001792811 | 1.014902402 | 0.012418635 |
| Grade | 2.075751122 | 1.813758443 | 2.375587961 | 2.70E-26 |
| riskScore | 1.311397517 | 1.218874164 | 1.410944213 | 3.81E-13 |
