# Supplementary material for: A Novel Necroptosis-Related lncRNA Signature for Predicting Prognosis and Immune Response of Glioma
Source: Biomed Res Int. 2022 Jun 16;2022:3742447. doi: 10.1155/2022/3742447 (PMC9226973; doi:10.1155/2022/3742447)
Supplement: Supplementary 6 — Table S6: the investigation of tumor microenvironment of the training cohort. [file 3742447.f6.docx]

Table S6 The investigation of tumor microenvironment of the training cohort

| ID | StromalScore | ImmuneScore | ESTIMATEScore | TumorPurity |
| --- | --- | --- | --- | --- |
| TCGA-41-2572 | -500.5633841 | 436.2280881 | -64.33529597 | 0.827843725 |
| TCGA-32-2634 | -875.2153504 | -482.9631678 | -1358.178518 | 0.918857002 |
| TCGA-06-0747 | -1350.775075 | -380.6238825 | -1731.398958 | 0.939085063 |
| TCGA-26-5134 | -850.0240214 | -61.29937941 | -911.3234008 | 0.891017147 |
| TCGA-32-1980 | 230.0016484 | 1195.671427 | 1425.673076 | 0.686407107 |
| TCGA-12-5299 | -532.3783407 | 455.6785711 | -76.69976961 | 0.82886048 |
| TCGA-06-2562 | -173.0276188 | 607.6340253 | 434.6064066 | 0.784577209 |
| TCGA-06-2559 | -591.4244736 | 126.1346342 | -465.2898394 | 0.859406766 |
| TCGA-76-4927 | 110.3527554 | 649.0219201 | 759.3746754 | 0.754138785 |
| TCGA-06-0158 | -501.6298715 | 631.3166603 | 129.6867888 | 0.811533953 |
| TCGA-19-2624 | -1513.923981 | -973.413763 | -2487.337744 | 0.97136771 |
| TCGA-12-3650 | -850.3743305 | 176.3132165 | -674.0611141 | 0.87466946 |
| TCGA-41-3915 | 230.2470458 | 1058.032168 | 1288.279213 | 0.700932918 |
| TCGA-12-0821 | -713.6693997 | -316.1956134 | -1029.865013 | 0.898781176 |
| TCGA-06-0125 | -534.09836 | -68.04021683 | -602.1385768 | 0.869503433 |
| TCGA-19-2625 | -840.9659534 | 75.03129626 | -765.9346571 | 0.881126642 |
| TCGA-32-5222 | -344.7124908 | 970.4184855 | 625.7059947 | 0.766878211 |
| TCGA-06-0139 | 1048.158635 | 2549.694173 | 3597.852808 | 0.423844793 |
| TCGA-06-2564 | -398.5076037 | 567.870217 | 169.3626133 | 0.808117244 |
| TCGA-06-0743 | -582.3226852 | 418.7035288 | -163.6191564 | 0.835930779 |
| TCGA-15-0742 | -1156.495618 | -367.0166317 | -1523.51225 | 0.928161827 |
| TCGA-06-0219 | -445.3373113 | 675.8437207 | 230.5064094 | 0.802798179 |
| TCGA-06-0129 | -1011.87929 | 856.3274794 | -155.5518108 | 0.835280267 |
| TCGA-28-1753 | 382.435143 | 1242.940466 | 1625.375609 | 0.664797597 |
| TCGA-06-0645 | 499.2245594 | 1734.598605 | 2233.823164 | 0.595517595 |
| TCGA-19-2619 | -704.4106079 | -290.1114366 | -994.5220445 | 0.896494712 |
| TCGA-08-0386 | -827.5362922 | -539.8113101 | -1367.347602 | 0.919387255 |
| TCGA-06-5408 | -783.5746888 | -10.94644322 | -794.521132 | 0.883103162 |
| TCGA-14-0736 | 252.2763311 | 1472.466674 | 1724.743005 | 0.653831196 |
| TCGA-14-1823 | -195.382664 | 1284.652431 | 1089.269767 | 0.721465894 |
| TCGA-02-0047 | -75.94544457 | 757.9559145 | 682.0104699 | 0.761547764 |
| TCGA-19-4065 | 179.1526323 | 1106.673175 | 1285.825807 | 0.701189728 |
| TCGA-14-1825 | -1023.768691 | -316.0120626 | -1339.780754 | 0.917788029 |
| TCGA-06-0171 | 1135.129916 | 2711.194818 | 3846.324734 | 0.390535552 |
| TCGA-06-5414 | -463.5723684 | 1004.278957 | 540.7065882 | 0.774825913 |
| TCGA-12-0619 | -217.4791896 | 1128.6866 | 911.2074105 | 0.739316309 |
| TCGA-06-0744 | -789.7631833 | 4.878361464 | -784.8848218 | 0.882438626 |
| TCGA-15-1444 | -377.082022 | 753.1976614 | 376.1156394 | 0.789871668 |
| TCGA-06-2565 | -901.1977187 | -28.64641984 | -929.8441386 | 0.892248028 |
| TCGA-06-5411 | -389.3987685 | 293.3557133 | -96.04305525 | 0.830445637 |
| TCGA-32-2616 | -720.4004075 | 328.9514999 | -391.4489076 | 0.853814497 |
| TCGA-76-4932 | -952.2769188 | 185.3254484 | -766.9514703 | 0.881197213 |
| TCGA-41-2571 | -1022.038904 | -149.8780576 | -1171.916962 | 0.907726411 |
| TCGA-27-2524 | -225.5132628 | 722.984248 | 497.4709852 | 0.778822369 |
| TCGA-14-0781 | 271.8432464 | 1033.116797 | 1304.960044 | 0.699184444 |
| TCGA-19-5960 | -1493.843 | -1048.794901 | -2542.637901 | 0.973264232 |
| TCGA-06-0211 | -285.2425128 | 718.1142891 | 432.8717763 | 0.784735058 |
| TCGA-27-1830 | 21.98763166 | 758.010945 | 779.9985767 | 0.752147226 |
| TCGA-06-0745 | -239.579498 | 740.0033207 | 500.4238226 | 0.778550421 |
| TCGA-32-2632 | -85.75824162 | 1291.166726 | 1205.408484 | 0.709556869 |
| TCGA-HW-7491 | -1616.208911 | -1112.998428 | -2729.207339 | 0.979188789 |
| TCGA-VM-A8CD | -827.2937558 | 218.3612608 | -608.932495 | 0.869995578 |
| TCGA-S9-A7QY | -1667.768365 | -1063.157287 | -2730.925652 | 0.979239948 |
| TCGA-FG-5965 | -1437.679876 | -684.2948264 | -2121.974702 | 0.95723546 |
| TCGA-HT-7475 | -1737.150662 | -1312.463879 | -3049.61454 | 0.987647708 |
| TCGA-HT-7473 | -404.4853114 | 1075.182417 | 670.6971059 | 0.762623008 |
| TCGA-DU-7292 | -1211.583209 | -1035.018823 | -2246.602032 | 0.962367585 |
| TCGA-DU-A5TT | -820.5121405 | -284.0749971 | -1104.587138 | 0.90353551 |
| TCGA-HT-7874 | -1407.010621 | -1239.503735 | -2646.514356 | 0.976653213 |
| TCGA-HT-7471 | -829.4054701 | 162.1912777 | -667.2141924 | 0.874181852 |
| TCGA-DB-5274 | -763.0894849 | 223.0200448 | -540.0694401 | 0.864967224 |
| TCGA-S9-A7R7 | -55.33950761 | 906.2985506 | 850.959043 | 0.74524234 |
| TCGA-S9-A6WG | 242.7800433 | 727.294186 | 970.0742294 | 0.733470315 |
| TCGA-HT-7691 | -406.2144534 | 482.5246148 | 76.31016133 | 0.816087069 |
| TCGA-HW-7487 | -1661.736127 | -1268.983777 | -2930.719903 | 0.984762816 |
| TCGA-E1-A7YJ | -1050.532591 | 475.4381774 | -575.0944134 | 0.867535811 |
| TCGA-E1-A7YK | -471.9124381 | 1054.318163 | 582.4057244 | 0.770941913 |
| TCGA-HT-7609 | -1256.557512 | -82.57326427 | -1339.130776 | 0.917750141 |
| TCGA-HT-A5RA | -708.1205814 | -40.11701566 | -748.2375971 | 0.879895258 |
| TCGA-S9-A6WL | -929.1788297 | -1011.051489 | -1940.230318 | 0.949177554 |
| TCGA-S9-A6TZ | -1016.529223 | -262.3077353 | -1278.836959 | 0.914199221 |
| TCGA-QH-A6XA | -1236.948732 | -442.5056061 | -1679.454338 | 0.936437238 |
| TCGA-HT-7858 | -938.6990566 | 590.8813629 | -347.8176937 | 0.850462942 |
| TCGA-TQ-A7RH | -927.2298768 | 182.7222232 | -744.5076537 | 0.879634966 |
| TCGA-DU-6403 | -1066.837137 | 202.2975961 | -864.539541 | 0.887878603 |
| TCGA-E1-A7YL | -427.6642511 | 839.2308213 | 411.5665702 | 0.786669651 |
| TCGA-FG-A4MX | -1516.44106 | -345.1479233 | -1861.588984 | 0.945481137 |
| TCGA-DB-A4XD | -1526.153588 | -708.6436855 | -2234.797273 | 0.96189525 |
| TCGA-VM-A8CF | -690.2007358 | 805.0095248 | 114.8087891 | 0.812808091 |
| TCGA-DU-6394 | -1560.23904 | -746.6609991 | -2306.900039 | 0.964735139 |
| TCGA-HT-7692 | -1547.88001 | -656.0640657 | -2203.944076 | 0.960647108 |
| TCGA-DH-5141 | -1287.636795 | -616.8630857 | -1904.49988 | 0.947513748 |
| TCGA-DB-A75L | -1259.232349 | -388.2114668 | -1647.443816 | 0.934778412 |
| TCGA-HT-8558 | -1425.249663 | -1497.868595 | -2923.118258 | 0.984568157 |
| TCGA-RY-A843 | -1441.044564 | -644.7929701 | -2085.837534 | 0.95568735 |
| TCGA-HT-A74O | -698.1586513 | 836.9029198 | 138.7442685 | 0.810756383 |
| TCGA-FG-A4MU | -467.8486885 | 571.1524572 | 103.3037687 | 0.813790713 |
| TCGA-DB-A64S | -652.4424717 | 496.0579276 | -156.384544 | 0.835347468 |
| TCGA-FG-A6J3 | -310.7956316 | 1065.408411 | 754.6127797 | 0.754597638 |
| TCGA-QH-A6X9 | -1512.99343 | -910.3558211 | -2423.349251 | 0.96909335 |
| TCGA-S9-A7R4 | -376.2681305 | 88.19526812 | -288.0728624 | 0.84581705 |
| TCGA-HT-7695 | -1505.219178 | -1291.196292 | -2796.415471 | 0.9811433 |
| TCGA-DU-A5TY | -242.5497636 | 1164.863232 | 922.3134686 | 0.738217591 |
| TCGA-S9-A89V | -1009.046511 | -236.0365937 | -1245.083104 | 0.912180051 |
| TCGA-FG-A4MT | -1481.342787 | -710.3818067 | -2191.724594 | 0.96014733 |
| TCGA-DU-7290 | -307.7798207 | 1038.011373 | 730.231552 | 0.756941219 |
| TCGA-DU-A5TW | -1591.535121 | -715.2900671 | -2306.825188 | 0.964732247 |
| TCGA-DB-5279 | -1367.974173 | -726.3577941 | -2094.331967 | 0.956053669 |
| TCGA-E1-A7YE | -354.2189622 | 905.027581 | 550.8086188 | 0.773887634 |
| TCGA-TM-A84F | -522.1018249 | 1321.290685 | 799.1888597 | 0.750287914 |
| TCGA-S9-A6WP | -1247.829726 | -44.64934963 | -1292.479076 | 0.915008932 |
| TCGA-DU-A6S2 | -1637.811534 | -1140.071811 | -2777.883346 | 0.980613886 |
| TCGA-QH-A6X8 | -1545.722882 | -749.9408742 | -2295.663756 | 0.964299679 |
| TCGA-DU-5854 | -340.5014432 | 389.5520453 | 49.05060203 | 0.818393047 |
| TCGA-TM-A84O | -1269.306964 | -544.0328464 | -1813.339811 | 0.943150863 |
| TCGA-DB-5278 | -1274.504065 | -934.3359679 | -2208.840033 | 0.960846486 |
| TCGA-HT-8109 | -1277.432467 | -789.0237327 | -2066.4562 | 0.954845975 |
| TCGA-DU-7294 | -1569.102461 | -1032.8469 | -2601.949361 | 0.975227041 |
| TCGA-FG-5962 | -1409.798715 | -1001.694835 | -2411.49355 | 0.968662566 |
| TCGA-S9-A7IX | -493.1331387 | 282.35263 | -210.7805087 | 0.839710159 |
| TCGA-HT-8110 | -380.820742 | 1334.427838 | 953.6070964 | 0.735111182 |
| TCGA-QH-A6X4 | -1848.736605 | -891.2496099 | -2739.986215 | 0.979508676 |
| TCGA-DB-A64V | -744.1786903 | 588.4617704 | -155.7169199 | 0.835293592 |
| TCGA-TQ-A7RK | -1403.680523 | -636.3690371 | -2040.04956 | 0.953687185 |
| TCGA-P5-A72U | 103.7683394 | 629.716249 | 733.4845884 | 0.756629089 |
| TCGA-TQ-A7RF | -1482.924623 | -432.709199 | -1915.633822 | 0.948035003 |
| TCGA-DU-5871 | -1261.461556 | -54.29872067 | -1315.760277 | 0.916382285 |
| TCGA-S9-A6TV | -390.8289194 | 942.4361603 | 551.6072409 | 0.773813385 |
| TCGA-TM-A7C5 | -1681.756304 | -1063.990634 | -2745.746939 | 0.979678633 |
| TCGA-RY-A83Z | -1680.576062 | -214.4193074 | -1894.995369 | 0.947066775 |
| TCGA-DU-7304 | -1506.932534 | -554.343958 | -2061.276492 | 0.954619807 |
| TCGA-DH-5144 | -1719.097957 | -972.0556749 | -2691.153632 | 0.978039864 |
| TCGA-DH-A7UT | -1270.005057 | -72.48883577 | -1342.493893 | 0.917946093 |
| TCGA-TQ-A8XE | -1344.911149 | -188.250913 | -1533.162062 | 0.928688077 |
| TCGA-DU-7006 | -466.0833002 | 786.9839797 | 320.9006795 | 0.79481619 |
| TCGA-P5-A733 | -1440.267359 | -908.5468337 | -2348.814192 | 0.96633635 |
| TCGA-FG-A87N | -1584.993255 | -739.4284738 | -2324.421729 | 0.965408951 |
| TCGA-DH-A66B | -902.2628654 | 579.460045 | -322.8028204 | 0.848525669 |
| TCGA-S9-A6TS | -1220.525395 | -242.5108154 | -1463.03621 | 0.924821418 |
| TCGA-DH-A669 | -1291.246148 | -297.7649317 | -1589.01108 | 0.931697157 |
| TCGA-E1-5302 | -911.1304565 | 282.8260485 | -628.3044079 | 0.871394109 |
| TCGA-S9-A7J3 | -1486.830118 | -526.3672599 | -2013.197378 | 0.952494149 |
| TCGA-HT-A617 | -376.5964929 | 620.0439143 | 243.4474213 | 0.801664099 |
| TCGA-DU-7011 | -165.4517449 | 603.8255054 | 438.3737604 | 0.78423421 |
| TCGA-FG-8181 | -1075.652923 | -1122.64774 | -2198.300663 | 0.960416676 |
| TCGA-FG-8182 | -1126.512788 | -8.563950324 | -1135.076738 | 0.905444274 |
| TCGA-TQ-A7RJ | -1198.705607 | -75.49091501 | -1274.196522 | 0.913922957 |
| TCGA-FG-5963 | -565.9540658 | -14.03033481 | -579.9844006 | 0.867892599 |
| TCGA-TM-A84J | -1244.090862 | -1175.470684 | -2419.561546 | 0.96895604 |
| TCGA-TQ-A7RN | -1712.269005 | -1035.743304 | -2748.012309 | 0.979745276 |
| TCGA-QH-A6XC | -1065.265705 | -412.253675 | -1477.51938 | 0.925628047 |
| TCGA-HW-8320 | -1206.07077 | -271.4328975 | -1477.503667 | 0.925627174 |
| TCGA-E1-5307 | -1419.364997 | -337.0044594 | -1756.369457 | 0.940338487 |
| TCGA-FG-7634 | -358.3798604 | 267.0285989 | -91.35126147 | 0.830061765 |
| TCGA-S9-A7J2 | -1741.441892 | -1186.486447 | -2927.928338 | 0.984691473 |
| TCGA-QH-A86X | -1641.730791 | -930.6118943 | -2572.342685 | 0.974256491 |
| TCGA-QH-A6CX | -843.8562289 | 175.9640631 | -667.8921657 | 0.874230173 |
| TCGA-HT-8013 | -805.9091139 | 1059.413127 | 253.5040135 | 0.800780796 |
| TCGA-HT-7610 | -1415.900521 | -1137.14246 | -2553.042981 | 0.97361391 |
| TCGA-S9-A6U2 | -1175.857925 | -581.1612013 | -1757.019127 | 0.940370929 |
| TCGA-QH-A6CS | -815.4540393 | -108.7411498 | -924.1951892 | 0.8918733 |
| TCGA-HT-8011 | -1401.683152 | -738.4045356 | -2140.087688 | 0.958001286 |
| TCGA-HT-7877 | -1515.232019 | -1070.537612 | -2585.769631 | 0.974698927 |
| TCGA-HT-7474 | -1346.210499 | -1002.461481 | -2348.67198 | 0.966330978 |
| TCGA-HT-8104 | -901.1954853 | -497.9644599 | -1399.159945 | 0.921214061 |
| TCGA-HT-8563 | 438.4668624 | 2083.674749 | 2522.141611 | 0.560995603 |
| TCGA-QH-A65R | -1522.568873 | -944.9448696 | -2467.513742 | 0.970672253 |
| TCGA-P5-A780 | -926.7405391 | -203.1389447 | -1129.879484 | 0.905120187 |
| TCGA-E1-A7YY | -1206.653796 | -1070.877043 | -2277.530838 | 0.96359141 |
| TCGA-DU-6393 | -1885.494796 | -1280.47311 | -3165.967906 | 0.990179704 |
| TCGA-R8-A73M | -1807.613982 | -1103.209605 | -2910.823587 | 0.984250725 |
| TCGA-HT-7879 | -1024.891144 | -294.6368311 | -1319.527975 | 0.916603534 |
| TCGA-FN-7833 | -378.4472326 | 1065.495984 | 687.0487517 | 0.761068239 |
| TCGA-IK-7675 | -1510.499136 | -1126.762273 | -2637.261409 | 0.976360536 |
| TCGA-TM-A7C3 | -1258.962444 | -856.568737 | -2115.531181 | 0.956961392 |
| TCGA-DU-8166 | -1218.574422 | -340.708651 | -1559.283073 | 0.930103228 |
| TCGA-DU-6406 | -1003.101013 | -129.3036515 | -1132.404665 | 0.905277717 |
| TCGA-E1-A7YV | -1658.449241 | -1217.195164 | -2875.644405 | 0.983324743 |
| TCGA-CS-5393 | -1100.835676 | 109.3756953 | -991.4599806 | 0.896295479 |
| TCGA-CS-6670 | -1544.542973 | -1139.765895 | -2684.308868 | 0.977829966 |
| TCGA-HT-7483 | -1062.299513 | -814.8995506 | -1877.199063 | 0.946224903 |
| TCGA-RY-A83X | -1708.642476 | -1125.967238 | -2834.609714 | 0.982211503 |
| TCGA-P5-A5F6 | -839.4367227 | -640.6009568 | -1480.037679 | 0.925767874 |
| TCGA-HT-7470 | -1198.14706 | -766.1696482 | -1964.316709 | 0.950284419 |
| TCGA-TM-A84Q | -1003.570092 | 287.4292959 | -716.1407961 | 0.877646778 |
| TCGA-R8-A6ML | -1865.128139 | -1082.403787 | -2947.531926 | 0.985188976 |
| TCGA-P5-A5EU | -671.3529602 | 516.9915246 | -154.3614357 | 0.835184181 |
| TCGA-DB-A4XG | -1996.416714 | -1574.457234 | -3570.873948 | 0.996735492 |
| TCGA-QH-A65X | -1085.428164 | -260.2224109 | -1345.650575 | 0.918129813 |
| TCGA-TQ-A7RS | -1769.975977 | -1176.061846 | -2946.037824 | 0.985151346 |
| TCGA-S9-A6U0 | 112.8154715 | 1053.604582 | 1166.420054 | 0.713577964 |
| TCGA-HT-8015 | -810.4283684 | -317.2868019 | -1127.71517 | 0.904985071 |
| TCGA-QH-A6X3 | -1398.46417 | -520.3852119 | -1918.849382 | 0.948185074 |
| TCGA-DU-6396 | -359.3319794 | 1420.466256 | 1061.134277 | 0.724319526 |
| TCGA-FG-A4MY | -1352.423631 | -535.279455 | -1887.703086 | 0.946722588 |
| TCGA-HT-7684 | -924.1701294 | 280.9092535 | -643.2608758 | 0.872469053 |
| TCGA-HT-7608 | -1118.345264 | -438.3910761 | -1556.73634 | 0.929965856 |
| TCGA-CS-5395 | -1167.722398 | -922.3092152 | -2090.031614 | 0.955868404 |
| TCGA-WY-A85E | -1767.290349 | -1080.323478 | -2847.613827 | 0.982568154 |
| TCGA-P5-A72X | -928.7197863 | -572.7324966 | -1501.452283 | 0.9269518 |
| TCGA-E1-5311 | -1636.9988 | -1288.201695 | -2925.200495 | 0.9846216 |
| TCGA-DU-5852 | -530.3015371 | 227.3299503 | -302.9715867 | 0.84698171 |
| TCGA-DB-5281 | -1031.76825 | -757.6768985 | -1789.445149 | 0.941979308 |
| TCGA-DU-A5TR | -384.6427481 | 1552.178049 | 1167.535301 | 0.713463266 |
| TCGA-CS-5396 | -1148.731128 | -570.6576804 | -1719.388808 | 0.938477704 |
| TCGA-HT-7620 | -1395.19959 | -469.1181526 | -1864.317743 | 0.945611511 |
| TCGA-HT-7856 | -1344.524845 | -1250.366924 | -2594.89177 | 0.974997354 |
| TCGA-HT-8019 | -1339.508346 | -1498.672295 | -2838.180642 | 0.982309795 |
| TCGA-HW-7493 | -604.5582063 | -97.33630289 | -701.8945092 | 0.876642536 |
| TCGA-QH-A65Z | -1695.611526 | -1000.116078 | -2695.727605 | 0.978179576 |
| TCGA-TM-A84T | -1308.241339 | -375.690919 | -1683.932258 | 0.936667642 |
| TCGA-P5-A5F1 | -955.0865798 | -86.78915517 | -1041.875735 | 0.899552689 |
| TCGA-06-5418 | -503.2862731 | 759.9068674 | 256.6205943 | 0.800506703 |
| TCGA-26-5135 | -151.0452385 | 618.7590227 | 467.7137841 | 0.781554742 |
| TCGA-28-1747 | -707.374084 | 941.1702886 | 233.7962046 | 0.802510154 |
| TCGA-06-0184 | 65.61719237 | 1367.540541 | 1433.157734 | 0.685607727 |
| TCGA-14-1402 | -510.490969 | 533.0793396 | 22.58837061 | 0.820619043 |
| TCGA-32-1970 | -828.5580006 | -109.4210571 | -937.9790577 | 0.892786588 |
| TCGA-12-3652 | -1214.308643 | -172.792377 | -1387.10102 | 0.920523946 |
| TCGA-06-0190 | 867.270139 | 1487.719023 | 2354.989161 | 0.581136105 |
| TCGA-76-4926 | -919.6758156 | -141.1017153 | -1060.777531 | 0.900761188 |
| TCGA-19-2629 | -557.3245779 | 687.4097353 | 130.0851574 | 0.811499784 |
| TCGA-27-1837 | -612.1495993 | 452.3236904 | -159.8259089 | 0.835625055 |
| TCGA-06-0878 | -198.687349 | 864.8975217 | 666.2101728 | 0.763048873 |
| TCGA-76-4931 | -1226.623697 | -406.5812654 | -1633.204963 | 0.934033902 |
| TCGA-06-0750 | 135.211972 | 881.5065353 | 1016.718507 | 0.728799215 |
| TCGA-28-2499 | -768.9549615 | 276.0687323 | -492.8862292 | 0.861470869 |
| TCGA-06-0649 | 259.8937574 | 1227.811263 | 1487.70502 | 0.679757013 |
| TCGA-06-0156 | -237.286934 | 979.033767 | 741.746833 | 0.75583555 |
| TCGA-28-5216 | -310.5403288 | 330.9969765 | 20.45664777 | 0.820797825 |
| TCGA-02-2486 | -3.995030345 | 1770.232983 | 1766.237953 | 0.649210416 |
| TCGA-02-0055 | 941.4984308 | 1491.762547 | 2433.260977 | 0.571747811 |
| TCGA-32-2615 | -175.9884548 | 810.1339379 | 634.1454831 | 0.766082558 |
| TCGA-06-0174 | -910.3805267 | -317.7457933 | -1228.12632 | 0.911157236 |
| TCGA-06-5856 | -286.2975581 | 389.4160859 | 103.1185278 | 0.813806515 |
| TCGA-06-0644 | 486.2078376 | 1138.98568 | 1625.193518 | 0.664817564 |
| TCGA-VM-A8CH | -1134.111457 | 38.5902014 | -1095.521255 | 0.902964459 |
| TCGA-FG-7641 | -1873.168198 | -1589.769095 | -3462.937293 | 0.995331269 |
| TCGA-DU-7299 | -1339.781695 | -252.0875486 | -1591.869243 | 0.931849469 |
| TCGA-HT-7485 | -1196.433972 | -390.641938 | -1587.07591 | 0.931593938 |
| TCGA-HT-7605 | -1311.04769 | -1038.039258 | -2349.086949 | 0.96634665 |
| TCGA-TQ-A7RM | -1678.168118 | -1515.397262 | -3193.565381 | 0.990737908 |
| TCGA-HT-A61B | -613.3565848 | 1044.527254 | 431.1706694 | 0.784889807 |
| TCGA-TQ-A7RV | -1223.043647 | -550.577016 | -1773.620663 | 0.941197054 |
| TCGA-S9-A6WO | -1291.358309 | 72.82517732 | -1218.533132 | 0.910576084 |
| TCGA-CS-6667 | -1206.467293 | -403.748897 | -1610.21619 | 0.932823273 |
| TCGA-DU-6397 | -1234.29827 | -610.2653511 | -1844.563621 | 0.944664278 |
| TCGA-S9-A7QW | -1848.236212 | -1161.312151 | -3009.548363 | 0.986709095 |
| TCGA-HT-7693 | -1101.959121 | 302.1937987 | -799.7653222 | 0.883464068 |
| TCGA-HW-7490 | -1076.732714 | -163.12909 | -1239.861804 | 0.91186571 |
| TCGA-CS-6666 | -863.9655995 | -334.8230062 | -1198.788606 | 0.909374287 |
| TCGA-E1-5305 | -1216.438893 | -463.1043286 | -1679.543222 | 0.936441816 |
| TCGA-DU-5870 | -1453.917332 | -709.1379362 | -2163.055268 | 0.958962628 |
| TCGA-DU-6407 | -1328.670212 | -239.5115834 | -1568.181795 | 0.93058221 |
| TCGA-S9-A7J1 | -1310.36521 | -645.6704066 | -1956.035617 | 0.949905209 |
| TCGA-FG-7637 | -1385.508953 | -958.3218867 | -2343.83084 | 0.96614789 |
| TCGA-CS-6186 | -1335.502834 | -555.7029189 | -1891.205753 | 0.946888045 |
| TCGA-S9-A7QX | -1423.74668 | -101.9292462 | -1525.675926 | 0.928279985 |
| TCGA-DB-A75O | -1052.245441 | 445.8391035 | -606.4063372 | 0.869812686 |
| TCGA-DB-A4XB | -1632.007967 | -740.6092911 | -2372.617258 | 0.967229396 |
| TCGA-FG-8189 | -1543.092976 | -1676.071716 | -3219.164692 | 0.99124116 |
| TCGA-HT-7606 | -1614.460105 | -844.3313787 | -2458.791484 | 0.970363659 |
| TCGA-DB-A4XA | -1098.701724 | -263.6435599 | -1362.345283 | 0.919098174 |
| TCGA-CS-5397 | -724.9487898 | -164.0280601 | -888.9768499 | 0.889523239 |
| TCGA-HW-A5KJ | -1596.961006 | -871.4301151 | -2468.391121 | 0.970703207 |
| TCGA-HT-7680 | -573.6604275 | 398.6139789 | -175.0464486 | 0.836850215 |
| TCGA-QH-A6CY | -1500.759839 | -1009.078798 | -2509.838636 | 0.97214711 |
| TCGA-DU-8167 | -639.8551513 | 235.0637807 | -404.7913706 | 0.854832419 |
| TCGA-DU-8162 | -1001.319173 | -851.6465487 | -1852.965721 | 0.94506814 |
| TCGA-DU-6400 | -1734.043634 | -984.8248707 | -2718.868505 | 0.978879658 |
| TCGA-TQ-A7RP | -766.4965189 | -312.1483474 | -1078.644866 | 0.901897174 |
| TCGA-WY-A859 | -1633.580702 | -1411.959743 | -3045.540445 | 0.987553825 |
| TCGA-P5-A5EW | -1163.374241 | -345.4763264 | -1508.850567 | 0.927358693 |
| TCGA-S9-A6UA | 208.5494019 | 1412.796932 | 1621.346334 | 0.665239309 |
| TCGA-FG-7636 | -1554.703895 | -528.579077 | -2083.282972 | 0.955576895 |
| TCGA-S9-A7IZ | -1094.509558 | -187.8123375 | -1282.321896 | 0.914406414 |
| TCGA-DU-A6S7 | -1212.669328 | 20.6300252 | -1192.039303 | 0.908961723 |
| TCGA-P5-A730 | -1322.90961 | -921.4626541 | -2244.372264 | 0.962278588 |
| TCGA-WH-A86K | -1266.022548 | -364.776042 | -1630.79859 | 0.933907677 |
| TCGA-TM-A84H | -1713.911961 | -888.278815 | -2602.190776 | 0.97523488 |
| TCGA-TQ-A7RW | -849.1574972 | 492.6283627 | -356.5291346 | 0.851134907 |
| TCGA-HT-7687 | -1324.623552 | -570.3745393 | -1894.998091 | 0.947066903 |
| TCGA-FG-A70Z | -211.5082912 | 312.8279291 | 101.3196379 | 0.813959939 |
| TCGA-FG-7643 | -1028.327423 | -863.1953443 | -1891.522768 | 0.946903007 |
| TCGA-F6-A8O3 | -1969.252303 | -1433.502717 | -3402.75502 | 0.994439799 |
| TCGA-CS-4938 | -1081.315756 | -386.0217921 | -1467.337549 | 0.925061414 |
| TCGA-WY-A85C | -1361.227706 | -403.9732581 | -1765.200964 | 0.940778771 |
| TCGA-HW-7486 | -1133.902849 | -392.7425069 | -1526.645356 | 0.928332895 |
| TCGA-HT-A74K | -702.7621452 | -135.8929651 | -838.6551103 | 0.886124117 |
| TCGA-DU-6404 | -1230.108477 | -883.3517072 | -2113.460184 | 0.956873123 |
| TCGA-HT-7602 | -1093.91885 | -165.2663334 | -1259.185183 | 0.91302637 |
| TCGA-E1-A7Z4 | -1376.084569 | 144.0787695 | -1232.0058 | 0.91139174 |
| TCGA-QH-A6CW | -1216.475833 | -32.35150001 | -1248.827333 | 0.912405138 |
| TCGA-S9-A7R2 | -493.4562271 | 757.6440041 | 264.187777 | 0.799840495 |
| TCGA-S9-A7IS | -1584.830837 | -686.3393054 | -2271.170142 | 0.963341345 |
| TCGA-DU-6410 | -1907.43488 | -1749.748764 | -3657.183644 | 0.997678342 |
| TCGA-DU-8164 | -1755.372633 | -1404.319337 | -3159.69197 | 0.990050495 |
| TCGA-FG-8191 | -1004.723045 | 51.53316098 | -953.1898836 | 0.893790181 |
| TCGA-FG-6688 | -651.5277537 | 575.2534437 | -76.27431008 | 0.828825539 |
| TCGA-DU-7298 | -1337.925213 | -379.5006926 | -1717.425906 | 0.938378162 |
| TCGA-E1-A7YM | -1371.210811 | -589.008715 | -1960.219526 | 0.950096975 |
| TCGA-DB-5270 | -651.0459754 | -249.1893422 | -900.2353177 | 0.890277086 |
| TCGA-TM-A7CA | -1660.327407 | -941.8899197 | -2602.217327 | 0.975235742 |
| TCGA-RY-A83Y | -1444.427059 | -224.5966505 | -1669.023709 | 0.935898979 |
| TCGA-DU-5855 | -1010.906137 | 282.8059785 | -728.1001587 | 0.878486848 |
| TCGA-HT-7603 | -1489.35596 | -1199.368966 | -2688.724927 | 0.977965499 |
| TCGA-DU-6402 | -457.7691031 | 687.0924556 | 229.3233526 | 0.802901712 |
| TCGA-TM-A7CF | -1765.444418 | -1162.218965 | -2927.663383 | 0.984684694 |
| TCGA-HT-7482 | -804.9910905 | 217.6967679 | -587.2943225 | 0.86842512 |
| TCGA-DB-A64Q | -1737.647859 | -930.7290132 | -2668.376872 | 0.977337582 |
| TCGA-DU-A76R | -1130.560928 | -950.7635669 | -2081.324495 | 0.955492122 |
| TCGA-DH-A66D | -1055.298399 | 211.2173159 | -844.0810828 | 0.886492958 |
| TCGA-DB-A75M | -1209.852044 | -238.0786368 | -1447.930681 | 0.923975675 |
| TCGA-DB-A4XF | -1100.792118 | -402.9952124 | -1503.787331 | 0.927080342 |
| TCGA-VM-A8CA | -795.8694037 | -47.2553086 | -843.1247123 | 0.886427988 |
| TCGA-DU-5847 | -599.4587631 | 722.001282 | 122.5425189 | 0.812146265 |
| TCGA-S9-A6UB | -1949.598278 | -1361.45455 | -3311.052827 | 0.992932237 |
| TCGA-S9-A6U6 | -1395.371249 | -572.9569809 | -1968.32823 | 0.95046761 |
| TCGA-DU-6399 | -894.4977224 | 116.0618855 | -778.4358369 | 0.881992907 |
| TCGA-E1-5322 | -1015.645835 | 380.8489162 | -634.7969187 | 0.871861252 |
| TCGA-E1-A7YO | -1539.287117 | -1248.610459 | -2787.897576 | 0.980900867 |
| TCGA-DU-7306 | -1086.673093 | -319.8310197 | -1406.504113 | 0.921632943 |
| TCGA-DH-A66G | -688.7195291 | -45.00740232 | -733.7269314 | 0.878881157 |
| TCGA-VM-A8C8 | -1177.724855 | -161.165372 | -1338.890227 | 0.917736117 |
| TCGA-S9-A7J0 | -1909.288588 | -1033.582848 | -2942.871436 | 0.985071441 |
| TCGA-W9-A837 | -1682.489939 | -1289.147619 | -2971.637558 | 0.985789549 |
| TCGA-FG-8185 | -1546.357594 | -708.488043 | -2254.845637 | 0.962695717 |
| TCGA-E1-5319 | -1185.989309 | -505.1525469 | -1691.141856 | 0.93703775 |
| TCGA-DU-8168 | -1971.33395 | -1265.472822 | -3236.806772 | 0.991579837 |
| TCGA-HT-7607 | -1129.490781 | -869.6911223 | -1999.181904 | 0.951865566 |
| TCGA-HT-8012 | -1511.480646 | -849.3547625 | -2360.835408 | 0.966788838 |
| TCGA-S9-A6WN | -538.9103764 | 783.5209644 | 244.610588 | 0.801562024 |
| TCGA-TM-A84M | -1936.921766 | -1278.443966 | -3215.365732 | 0.991167361 |
| TCGA-DB-A4X9 | -1618.887074 | -621.0997186 | -2239.986793 | 0.96210325 |
| TCGA-RY-A845 | -1470.736344 | -599.2464694 | -2069.982814 | 0.954999647 |
| TCGA-DU-7018 | -1659.74223 | -1348.703262 | -3008.445492 | 0.986682775 |
| TCGA-HW-A5KL | -1321.408067 | -292.7941451 | -1614.202212 | 0.933033946 |
| TCGA-S9-A6U1 | -1251.564564 | -395.7508023 | -1647.315367 | 0.934771714 |
| TCGA-HT-7476 | -589.6304157 | -617.1373876 | -1206.767803 | 0.90986088 |
| TCGA-CS-4944 | -967.0499751 | -91.92376554 | -1058.973741 | 0.900646161 |
| TCGA-DU-6395 | -1223.548903 | -244.3622127 | -1467.911115 | 0.925093389 |
| TCGA-FG-7638 | -1421.570913 | -843.8253418 | -2265.396255 | 0.963113622 |
| TCGA-HT-8111 | -1043.034605 | 239.1850456 | -803.8495594 | 0.883744783 |
| TCGA-P5-A781 | -1444.64555 | -896.5062309 | -2341.151781 | 0.96604636 |
| TCGA-DU-7007 | -296.7465802 | 458.7294235 | 161.9828433 | 0.808754836 |
| TCGA-HT-7884 | -1551.65144 | -1014.043766 | -2565.695207 | 0.974036046 |
| TCGA-FG-6692 | -954.5452933 | 156.3468099 | -798.1984833 | 0.883356293 |
| TCGA-DU-A7TG | -1027.632131 | -709.0270762 | -1736.659208 | 0.939350157 |
| TCGA-KT-A74X | -1667.180582 | -604.4031228 | -2271.583705 | 0.963357629 |
| TCGA-DB-A64X | -1426.854194 | -704.2822195 | -2131.136414 | 0.957623668 |
| TCGA-E1-A7YQ | 19.06794855 | 1213.349071 | 1232.41702 | 0.706757695 |
| TCGA-WY-A85B | -1047.760641 | 294.165056 | -753.5955849 | 0.880268701 |
| TCGA-HT-7854 | -506.1915791 | 512.6694745 | 6.477895453 | 0.821968191 |
| TCGA-HT-7676 | -1056.928098 | 400.384211 | -656.543887 | 0.873420198 |
| TCGA-DB-A64W | -1965.63788 | -1953.990808 | -3919.628689 | 0.999561029 |
| TCGA-P5-A5F0 | -1975.340935 | -1592.403599 | -3567.744534 | 0.996698299 |
| TCGA-HW-8322 | -1599.534327 | -723.9146291 | -2323.448956 | 0.965371709 |
| TCGA-HT-A618 | -702.1796615 | 1049.906405 | 347.7267433 | 0.792420407 |
| TCGA-R8-A6MO | -1445.708815 | -328.9877894 | -1774.696604 | 0.941250402 |
| TCGA-DU-7010 | -1040.988372 | -771.0872553 | -1812.075627 | 0.943089171 |
| TCGA-RY-A840 | -1843.534554 | -974.9448785 | -2818.479433 | 0.981764141 |
| TCGA-DU-A7T8 | -773.4151377 | 118.3427403 | -655.0723974 | 0.873314993 |
| TCGA-DU-7015 | -973.0216942 | 536.7809967 | -436.2406975 | 0.857218769 |
| TCGA-HW-A5KK | -442.5773475 | 884.892198 | 442.3148504 | 0.783875135 |
| TCGA-CS-4941 | -700.0223965 | 707.4565359 | 7.434139387 | 0.82188824 |
| TCGA-DU-5872 | -51.2941995 | 1406.839237 | 1355.545038 | 0.693856571 |
| TCGA-HT-A4DV | -1297.044945 | -538.6236873 | -1835.668632 | 0.944235159 |
| TCGA-DU-8165 | -244.9941051 | 478.6199324 | 233.6258273 | 0.802525075 |
| TCGA-P5-A5EZ | -1064.455388 | 42.23173717 | -1022.223651 | 0.898288876 |
| TCGA-EZ-7264 | -1830.992938 | -1375.2616 | -3206.254537 | 0.990989111 |
| TCGA-DU-6408 | -1287.790457 | -181.9776743 | -1469.768132 | 0.925196867 |
| TCGA-S9-A6WH | -1762.887267 | -1006.730594 | -2769.617861 | 0.980375422 |
| TCGA-TM-A84C | -757.8466299 | 152.8055299 | -605.0411 | 0.869713795 |
| TCGA-S9-A6TW | -1000.688808 | -565.0112619 | -1565.70007 | 0.930448789 |
| TCGA-DU-6542 | -948.7460579 | 228.7149954 | -720.0310624 | 0.877920342 |
| TCGA-VM-A8C9 | -244.6305929 | 1194.863117 | 950.2325241 | 0.735446912 |
| TCGA-E1-A7YN | 229.0306087 | 1915.105102 | 2144.135711 | 0.606041746 |
| TCGA-DU-6392 | -533.9574858 | 54.62470379 | -479.332782 | 0.860458888 |
| TCGA-HT-7472 | -820.6583857 | 260.8587627 | -559.7996231 | 0.86641697 |
| TCGA-DU-A7TD | -557.1778912 | 789.3679354 | 232.1900442 | 0.802650799 |
| TCGA-CS-6188 | -695.394739 | 138.320414 | -557.074325 | 0.866217151 |
| TCGA-TM-A84I | -1533.101542 | -585.6892811 | -2118.790823 | 0.957100144 |
| TCGA-DU-5849 | -1450.561375 | -828.9628174 | -2279.524192 | 0.963669604 |
| TCGA-HT-7467 | -1229.911543 | -962.2941899 | -2192.205733 | 0.960167067 |
| TCGA-P5-A5EV | -1361.399494 | -332.0907674 | -1693.490262 | 0.93715808 |
| TCGA-DH-A66F | -1640.829331 | -916.109493 | -2556.938824 | 0.973744251 |
| TCGA-S9-A6WM | -794.587116 | -580.6247257 | -1375.211842 | 0.919840721 |
| TCGA-P5-A5EX | -271.3593938 | 1052.389461 | 781.0300672 | 0.752047438 |
| TCGA-HT-7480 | -1627.180352 | -1216.686496 | -2843.866848 | 0.982465756 |
| TCGA-IK-8125 | -1376.880862 | -865.3078453 | -2242.188707 | 0.962191336 |
| TCGA-HT-7881 | -1674.542886 | -1431.39917 | -3105.942055 | 0.988909489 |
| TCGA-CS-4943 | -1231.538742 | -416.5610252 | -1648.099767 | 0.934812612 |
| TCGA-P5-A77X | -1773.057668 | -1395.199193 | -3168.25686 | 0.99022662 |
| TCGA-DB-5275 | -1508.575906 | -532.4392272 | -2041.015133 | 0.953729809 |
| TCGA-P5-A737 | -1556.25352 | -756.6115958 | -2312.865116 | 0.964965248 |
| TCGA-HT-8564 | -992.8703879 | -460.907022 | -1453.77741 | 0.924303566 |
| TCGA-DU-A7TA | -875.1885856 | 221.9099986 | -653.278587 | 0.87318669 |
| TCGA-HT-7902 | -1363.586623 | -613.733359 | -1977.319982 | 0.950877034 |
| TCGA-VM-A8CE | -1272.002991 | -763.5534564 | -2035.556447 | 0.953488588 |
| TCGA-HW-7495 | -1497.710451 | -1246.529796 | -2744.240247 | 0.979634249 |
| TCGA-FG-6689 | -1114.591953 | -22.53011129 | -1137.122065 | 0.90557167 |
| TCGA-HT-A61C | -838.3494971 | 109.6215497 | -728.7279474 | 0.878530872 |
| TCGA-E1-A7YS | -2052.771482 | -1234.090471 | -3286.861952 | 0.992504543 |
| TCGA-WY-A85A | -1545.262396 | -471.8920569 | -2017.154453 | 0.952670891 |
| TCGA-HT-7873 | -1251.341977 | -409.2854825 | -1660.627459 | 0.935464107 |
| TCGA-R8-A6YH | -1453.734296 | -649.9081591 | -2103.642455 | 0.956453473 |
| TCGA-DU-A7T6 | -1126.351186 | -941.2378908 | -2067.589077 | 0.954895368 |
| TCGA-P5-A731 | -755.3526098 | -422.763124 | -1178.115734 | 0.908107799 |
| TCGA-TQ-A7RQ | -1886.84867 | -1583.344803 | -3470.193473 | 0.995433508 |
| TCGA-TM-A84R | -1454.567165 | -1475.982225 | -2930.54939 | 0.984758463 |
| TCGA-DU-A5TP | -823.0318582 | 1137.690372 | 314.6585138 | 0.7953719 |
| TCGA-DU-A76L | 102.437684 | 571.3305645 | 673.7682485 | 0.762331329 |
| TCGA-HT-7468 | -1559.255858 | -850.1452352 | -2409.401093 | 0.96858623 |
| TCGA-DU-A7TC | -1570.219097 | -972.943813 | -2543.16291 | 0.97328193 |
| TCGA-DB-5277 | -1398.255915 | -1037.66423 | -2435.920145 | 0.969546915 |
| TCGA-DU-7014 | -1105.517071 | -354.7743102 | -1460.291381 | 0.924668076 |
| TCGA-DB-A75P | -890.7099864 | -421.8588241 | -1312.568811 | 0.916194653 |
| TCGA-FG-6691 | -635.7646709 | 676.1311296 | 40.36645875 | 0.819124918 |
| TCGA-P5-A735 | -1618.361735 | -538.0006037 | -2156.362339 | 0.958683611 |
| TCGA-DB-A4XC | -1230.942648 | -56.26329904 | -1287.205947 | 0.914696387 |
| TCGA-CS-6668 | -2048.48804 | -1390.240043 | -3438.728084 | 0.994981997 |
| TCGA-HT-7601 | -191.8674115 | 1252.039346 | 1060.171934 | 0.724416913 |
| TCGA-WY-A858 | -339.8035421 | 804.3285545 | 464.5250123 | 0.781846658 |
| TCGA-HT-7860 | -656.8791725 | 434.5641582 | -222.3150143 | 0.840628384 |
| TCGA-S9-A6WQ | -948.1578316 | 307.0910717 | -641.0667599 | 0.872311622 |
| TCGA-HT-8114 | -482.6714965 | 633.1792919 | 150.5077953 | 0.809744364 |
| TCGA-FG-A60K | -1667.383534 | -612.8402872 | -2280.223821 | 0.963697029 |
| TCGA-HT-7479 | -681.8375568 | 174.3866482 | -507.4509086 | 0.862554553 |
| TCGA-DU-5874 | -1731.252215 | -1351.941178 | -3083.193394 | 0.988408034 |
| TCGA-HT-8113 | -1559.799483 | -1382.097243 | -2941.896725 | 0.985046801 |
| TCGA-DU-7008 | -1755.789808 | -1096.496571 | -2852.28638 | 0.982695429 |
| TCGA-DH-5140 | -1193.141485 | -876.7262935 | -2069.867778 | 0.954994638 |
| TCGA-HT-7855 | -1462.428725 | -601.6011611 | -2064.029886 | 0.954740101 |
| TCGA-P5-A5F4 | -1487.080804 | -1285.178732 | -2772.259535 | 0.980451793 |
| TCGA-DU-7019 | -826.3257019 | -245.7401184 | -1072.06582 | 0.901479607 |
| TCGA-HT-A615 | -1135.182952 | 224.8125483 | -910.3704041 | 0.890953633 |
| TCGA-DH-5142 | -626.8084277 | 1003.136289 | 376.327861 | 0.789852564 |
| TCGA-DU-A7TB | -1315.228091 | -914.252352 | -2229.480443 | 0.961681568 |
| TCGA-HT-7880 | -1239.368127 | -831.5334208 | -2070.901547 | 0.955039638 |
| TCGA-KT-A7W1 | -1366.243393 | -577.8804516 | -1944.123845 | 0.949357281 |
| TCGA-HT-A4DS | -938.3849317 | 682.4761795 | -255.9087522 | 0.843288936 |
| TCGA-DU-A7TJ | -966.0982858 | -117.0544097 | -1083.152695 | 0.902182797 |
| TCGA-CS-5394 | -1890.60364 | -1426.128319 | -3316.731959 | 0.99303083 |
| TCGA-E1-5304 | -2110.892962 | -1557.739391 | -3668.632353 | 0.997791382 |
| TCGA-QH-A65V | -1419.989757 | -460.2447869 | -1880.234544 | 0.946368957 |
| TCGA-HT-A5R7 | -1348.019161 | -845.881393 | -2193.900554 | 0.960236553 |
| TCGA-S9-A6TU | -915.5803346 | 191.5359544 | -724.0443801 | 0.878202258 |
| TCGA-FG-A60L | -836.5434217 | 439.5840072 | -396.9594145 | 0.854235301 |
| TCGA-HW-8319 | -1414.595315 | -880.5418131 | -2295.137128 | 0.964279206 |
| TCGA-FG-A4MW | -1121.727715 | -556.4689789 | -1678.196693 | 0.936372456 |
| TCGA-P5-A5ET | -1667.260133 | -1337.978251 | -3005.238385 | 0.986606093 |
| TCGA-HT-7686 | -605.9668833 | 1053.258903 | 447.2920202 | 0.783421289 |
| TCGA-DU-7302 | -1760.319204 | -1381.164409 | -3141.483613 | 0.989670866 |
| TCGA-DB-5273 | -583.3989301 | 346.7882324 | -236.6106977 | 0.841763074 |
| TCGA-S9-A6U8 | -1028.73674 | 90.20060526 | -938.5361346 | 0.892823422 |
| TCGA-HT-8107 | -1241.399996 | -1298.098813 | -2539.498809 | 0.973158292 |
| TCGA-FG-5964 | -1029.421562 | 209.5187514 | -819.9028107 | 0.884845066 |
| TCGA-DU-7300 | -1089.842041 | -664.1296758 | -1753.971717 | 0.940218677 |
| TCGA-VW-A7QS | -1653.324504 | -1044.841038 | -2698.165542 | 0.978253863 |
| TCGA-TQ-A7RO | -1695.392661 | -1014.362646 | -2709.755307 | 0.978605304 |
| TCGA-DU-8158 | -376.8693164 | 697.5595859 | 320.6902695 | 0.794834933 |
| TCGA-TM-A84G | -1919.584682 | -1457.544017 | -3377.128699 | 0.994036638 |
| TCGA-HT-8010 | -1100.134371 | -719.4909953 | -1819.625366 | 0.943457117 |
| TCGA-HT-7690 | -494.5782715 | 582.8206745 | 88.24240298 | 0.815073567 |
| TCGA-DU-A76K | -1300.046232 | -1437.552109 | -2737.598341 | 0.979438022 |
| TCGA-DH-5143 | -1046.070051 | -245.6243964 | -1291.694447 | 0.914962461 |
| TCGA-HT-7477 | -1266.037708 | -719.6807708 | -1985.718479 | 0.951257948 |
| TCGA-VW-A8FI | -1115.230591 | 237.6178357 | -877.6127556 | 0.888759855 |
| TCGA-E1-A7YI | -1625.827353 | -816.3814086 | -2442.208762 | 0.969772574 |
| TCGA-FG-6690 | -1112.064693 | 39.85448363 | -1072.21021 | 0.90148878 |
| TCGA-DH-A7US | -1531.76177 | -737.7500824 | -2269.511852 | 0.963276012 |
| TCGA-HT-7689 | -1259.374006 | -276.6693246 | -1536.04333 | 0.928844845 |
| TCGA-P5-A5EY | -1042.919449 | -547.3513225 | -1590.270771 | 0.931764306 |
| TCGA-CS-5390 | -1645.068361 | -1207.766953 | -2852.835314 | 0.982710351 |
| TCGA-QH-A6CV | -884.5016241 | 333.3963385 | -551.1052856 | 0.865779017 |
| TCGA-HT-8105 | -1519.006267 | -764.8685625 | -2283.87483 | 0.963839982 |
| TCGA-S9-A7R1 | -1669.07086 | -1238.143265 | -2907.214126 | 0.984156925 |
| TCGA-HT-7681 | -1219.058346 | -547.2451196 | -1766.303466 | 0.940833624 |
| TCGA-TM-A84B | -1142.482738 | -1030.998565 | -2173.481303 | 0.959395429 |
| TCGA-HW-7489 | -1296.54897 | -839.3873523 | -2135.936322 | 0.957826362 |
| TCGA-CS-6669 | -1501.027094 | -1642.317078 | -3143.344171 | 0.989709982 |
| TCGA-S9-A7R3 | -1065.249368 | -84.7900529 | -1150.039421 | 0.906374363 |
| TCGA-HT-7882 | 1499.404235 | 1988.068482 | 3487.472718 | 0.438463702 |
| TCGA-DB-A75K | -956.1275732 | 133.012222 | -823.1153512 | 0.885064662 |
| TCGA-HT-A61A | -1359.093621 | -1186.923278 | -2546.016899 | 0.973378037 |
| TCGA-E1-A7Z2 | -1410.048796 | -1096.724169 | -2506.772965 | 0.972041543 |
| TCGA-06-5412 | 554.8908501 | 1760.219125 | 2315.109976 | 0.585889982 |
| TCGA-12-5295 | -447.3799528 | 1030.679211 | 583.2992579 | 0.77085837 |
| TCGA-27-1834 | -402.5637656 | 916.4800407 | 513.9162751 | 0.777305947 |
| TCGA-27-1831 | 21.07099879 | 1028.226895 | 1049.297894 | 0.725516338 |
| TCGA-06-0132 | 432.1138905 | 1601.671452 | 2033.785343 | 0.618846221 |
| TCGA-12-0618 | -1244.112177 | -654.0470501 | -1898.159227 | 0.947215768 |
| TCGA-28-5208 | -303.1927048 | 436.8483112 | 133.6556064 | 0.811193413 |
| TCGA-06-5417 | -1125.537068 | -487.5670554 | -1613.104124 | 0.932975941 |
| TCGA-28-5215 | 194.3458932 | 690.9687913 | 885.3146845 | 0.741870231 |
| TCGA-14-0790 | -1096.543724 | -703.4680305 | -1800.011755 | 0.942498819 |
| TCGA-28-2509 | -840.0476028 | 420.7299281 | -419.3176747 | 0.855936931 |
| TCGA-06-0210 | -76.26977952 | 1334.68663 | 1258.416851 | 0.704052568 |
| TCGA-12-0616 | -713.1645673 | 473.5654622 | -239.5991051 | 0.841999804 |
| TCGA-76-4929 | -680.7720154 | 123.8015095 | -556.9705059 | 0.866209537 |
| TCGA-06-2567 | -275.1979125 | 1003.824958 | 728.6270456 | 0.757095108 |
| TCGA-14-0789 | -40.36553058 | 1376.537601 | 1336.17207 | 0.695901568 |
| TCGA-28-5213 | 563.7347503 | 1804.755466 | 2368.490216 | 0.579522164 |
| TCGA-06-2569 | -255.2828571 | -1243.36425 | -1498.647107 | 0.926797234 |
| TCGA-06-0882 | -130.0225805 | 1070.987507 | 940.9649269 | 0.736367997 |
| TCGA-27-1832 | -51.66415883 | 1269.094938 | 1217.43078 | 0.708312249 |
| TCGA-26-1442 | -1000.446573 | -207.228932 | -1207.675505 | 0.909916155 |
| TCGA-14-2554 | -301.1983621 | 903.4578541 | 602.2594919 | 0.769082506 |
| TCGA-16-1045 | 478.9486787 | 1612.672454 | 2091.621132 | 0.612155298 |
| TCGA-06-2561 | -451.91709 | 1044.016822 | 592.0997318 | 0.770034837 |
| TCGA-06-0749 | -487.4386119 | 979.2796615 | 491.8410496 | 0.779340463 |
| TCGA-76-4928 | -299.5777989 | 843.8609393 | 544.2831403 | 0.774493917 |
| TCGA-14-0787 | -804.4760347 | 583.5628287 | -220.913206 | 0.840516919 |
| TCGA-06-0152 | -205.6113319 | 1391.897611 | 1186.28628 | 0.711531961 |
| TCGA-06-5410 | 777.0994897 | 2385.805637 | 3162.905127 | 0.480768855 |
| TCGA-26-5136 | -126.4077337 | 981.5214009 | 855.1136672 | 0.744835557 |
| TCGA-41-5651 | -1111.644349 | -810.2985489 | -1921.942898 | 0.94832925 |
| TCGA-19-1389 | 707.1827754 | 1309.417739 | 2016.600515 | 0.620825731 |
| TCGA-26-5132 | -581.7782752 | 254.046822 | -327.7314532 | 0.848908272 |
| TCGA-06-0646 | -158.4401352 | 970.0442772 | 811.604142 | 0.749081846 |
| TCGA-19-2620 | -606.9609733 | 137.9254745 | -469.0354988 | 0.859687755 |
| TCGA-27-1835 | -1054.415265 | -697.3426715 | -1751.757937 | 0.940107957 |
| TCGA-76-4925 | -1101.350872 | -547.1866523 | -1648.537524 | 0.93483543 |
| TCGA-06-2557 | -148.6512085 | 1667.840057 | 1519.188848 | 0.676360214 |
| TCGA-14-1034 | -1086.829987 | 39.22268597 | -1047.607301 | 0.899919872 |
| TCGA-27-2519 | -71.94941848 | 1205.402851 | 1133.453432 | 0.716959764 |
| TCGA-26-5139 | -468.7714622 | 381.7715125 | -86.99994965 | 0.829705399 |
| TCGA-28-2510 | -312.4906962 | -130.0847797 | -442.5754759 | 0.857697239 |
| TCGA-06-0221 | -1062.188959 | -1032.650514 | -2094.839473 | 0.956075508 |
| TCGA-27-2526 | -831.3074892 | 843.677638 | 12.37014886 | 0.821475285 |
| TCGA-06-5858 | -250.4020666 | 479.2322428 | 228.8301763 | 0.802944864 |
| TCGA-16-0846 | -397.3893506 | 1009.459932 | 612.0705817 | 0.768161235 |
| TCGA-06-0157 | -320.2645437 | 468.9081532 | 148.6436095 | 0.809904901 |
| TCGA-28-5209 | -808.3258923 | -299.3597258 | -1107.685618 | 0.903730313 |
| TCGA-41-4097 | 231.990994 | 898.8032976 | 1130.794292 | 0.717231815 |
| TCGA-14-0817 | 22.29381863 | 1402.851548 | 1425.145367 | 0.686463436 |
| TCGA-32-4213 | 397.0289101 | 1366.554574 | 1763.583484 | 0.649506735 |
| TCGA-28-5207 | -165.2447859 | 174.6068232 | 9.362037258 | 0.821727 |
| TCGA-06-5859 | -455.6548054 | 795.4149014 | 339.760096 | 0.793133177 |
| TCGA-06-2563 | -634.31341 | 134.2941344 | -500.0192756 | 0.862002095 |
| TCGA-14-0871 | -684.8879588 | -876.3155735 | -1561.203532 | 0.930206733 |
| TCGA-06-0686 | -539.8655274 | 193.5941738 | -346.2713536 | 0.850343518 |
| TCGA-FG-A710 | -915.8438122 | 483.6214466 | -432.2223655 | 0.856914879 |
| TCGA-FG-A6IZ | -1517.823564 | -428.7651751 | -1946.588739 | 0.949470902 |
| TCGA-S9-A7QZ | -1761.47136 | -1414.771203 | -3176.242563 | 0.990389425 |
| TCGA-HT-A5RB | -1582.655962 | -1303.493934 | -2886.149896 | 0.983604014 |
| TCGA-DH-A7UV | -1280.438793 | -193.2449743 | -1473.683767 | 0.925414832 |
| TCGA-E1-A7YH | -1404.469666 | -258.1877918 | -1662.657458 | 0.935569378 |
| TCGA-DB-A64P | -1238.272832 | -564.819232 | -1803.092064 | 0.942649836 |
| TCGA-DH-A7UR | -1301.103433 | -1184.433749 | -2485.537182 | 0.971304883 |
| TCGA-DB-A64O | -896.412579 | -347.1801033 | -1243.592682 | 0.912090377 |
| TCGA-DH-A7UU | -1428.564022 | -683.4333483 | -2111.997371 | 0.956810723 |
| TCGA-FG-A60J | -1137.047415 | -259.660077 | -1396.707492 | 0.921073944 |
| TCGA-DU-A6S6 | -1752.345163 | -1568.941774 | -3321.286937 | 0.993109407 |
| TCGA-VV-A829 | -1293.166677 | -247.4416782 | -1540.608355 | 0.929092885 |
| TCGA-DB-A64L | -1561.836565 | -514.1127685 | -2075.949334 | 0.955259054 |
| TCGA-DU-6401 | -802.1145353 | -273.1049349 | -1075.21947 | 0.901679871 |
| TCGA-QH-A6CU | -1026.428489 | -79.25288333 | -1105.681372 | 0.903604327 |
| TCGA-DU-7012 | 435.0647777 | 1583.852414 | 2018.917191 | 0.620559105 |
| TCGA-CS-4942 | -868.7398404 | 770.5516805 | -98.18815991 | 0.830621013 |
| TCGA-HW-A5KM | -259.783241 | 1793.048878 | 1533.265637 | 0.674836788 |
| TCGA-S9-A6WE | -1501.92547 | -757.3525005 | -2259.277971 | 0.962871561 |
| TCGA-P5-A736 | -476.8003727 | 767.5053473 | 290.7049746 | 0.797498164 |
| TCGA-CS-6290 | -593.1908292 | 1085.950657 | 492.7598279 | 0.779255949 |
| TCGA-HT-A616 | -1357.926139 | -1058.627636 | -2416.553776 | 0.968846791 |
| TCGA-TM-A84S | -1337.630549 | -484.5899109 | -1822.22046 | 0.943583325 |
| TCGA-RY-A847 | -1031.755453 | -62.10027515 | -1093.855728 | 0.902859375 |
| TCGA-QH-A65S | -1206.817305 | -58.47956993 | -1265.296875 | 0.91339194 |
| TCGA-S9-A7IY | -1137.270476 | 189.2925392 | -947.9779365 | 0.893446804 |
| TCGA-DU-A5TU | -729.7916735 | 720.7019846 | -9.089688905 | 0.823267507 |
| TCGA-DU-7301 | -728.7617855 | 199.2315243 | -529.5302612 | 0.864189846 |
| TCGA-DB-A4XH | -1549.585801 | -1351.513832 | -2901.099633 | 0.983997396 |
| TCGA-HT-7478 | -323.3097924 | 1290.493682 | 967.1838896 | 0.733758633 |
| TCGA-S9-A89Z | -611.194473 | 600.0808948 | -11.11357823 | 0.823436111 |
| TCGA-HT-A74L | -1461.084299 | -377.5249337 | -1838.609233 | 0.9443772 |
| TCGA-HT-8018 | -34.23627012 | 355.1085651 | 320.8722949 | 0.794818719 |
| TCGA-P5-A77W | -1783.513801 | -1229.911431 | -3013.425232 | 0.986801408 |
| TCGA-DB-5280 | -1235.661379 | -740.4074316 | -1976.068811 | 0.950820163 |
| TCGA-P5-A5F2 | -1584.349686 | -1130.207766 | -2714.557452 | 0.978750092 |
| TCGA-FG-A6J1 | -1075.28491 | -74.84234143 | -1150.127251 | 0.906379809 |
| TCGA-HT-A5RC | -905.3922452 | -16.85624201 | -922.2484872 | 0.891744022 |
| TCGA-FG-A70Y | -991.9310531 | 146.0108857 | -845.9201674 | 0.886617846 |
| TCGA-HT-7604 | -1412.639346 | -832.9898972 | -2245.629244 | 0.962328771 |
| TCGA-HT-7694 | -1568.509649 | -1373.54054 | -2942.050189 | 0.985050681 |
| TCGA-TQ-A7RI | -1552.222258 | -605.7665394 | -2157.988797 | 0.9587515 |
| TCGA-DU-8161 | -359.3135326 | 918.1461325 | 558.8325999 | 0.773141152 |
| TCGA-VV-A86M | -1292.119526 | -2.896283002 | -1295.015809 | 0.915159093 |
| TCGA-E1-5303 | -661.9752129 | 849.3902449 | 187.415032 | 0.806553569 |
| TCGA-E1-A7Z3 | -930.68257 | -287.0229054 | -1217.705475 | 0.91052586 |
| TCGA-DB-A64U | -1489.260446 | -952.9323636 | -2442.192809 | 0.969772002 |
| TCGA-TM-A7C4 | -1590.541782 | -1345.545587 | -2936.087369 | 0.984899525 |
| TCGA-DU-6405 | -1291.775664 | -265.9798511 | -1557.755516 | 0.930020847 |
| TCGA-DU-A7TI | -1010.435604 | 355.0263711 | -655.4092324 | 0.873339079 |
| TCGA-FG-A713 | -1318.343858 | -1348.07959 | -2666.423448 | 0.977276843 |
| TCGA-F6-A8O4 | -1631.67561 | -1181.646656 | -2813.322266 | 0.981619949 |
| TCGA-E1-A7YD | -1247.307604 | -491.1905274 | -1738.498132 | 0.939442698 |
| TCGA-P5-A72W | -1482.757764 | -518.3448446 | -2001.102609 | 0.951951946 |
| TCGA-FG-8186 | -1047.327195 | 182.11102 | -865.2161751 | 0.887924295 |
| TCGA-DU-A6S8 | -1654.695941 | -1080.721763 | -2735.417704 | 0.979373395 |
| TCGA-P5-A72Z | -1806.11938 | -1241.672395 | -3047.791774 | 0.987605748 |
| TCGA-HW-8321 | -1141.077925 | -278.1491211 | -1419.227046 | 0.922356073 |
| TCGA-HT-A619 | -1824.055321 | -1286.990406 | -3111.045727 | 0.989020476 |
| TCGA-TQ-A7RR | -1119.774502 | -115.5546635 | -1235.329166 | 0.911592394 |
| TCGA-HT-7857 | 803.5990659 | 2076.358174 | 2879.95724 | 0.516762287 |
| TCGA-S9-A6U9 | -466.0924706 | 584.9250706 | 118.8326 | 0.812463878 |
| TCGA-E1-A7YU | -1369.103946 | -579.1962565 | -1948.300202 | 0.949549719 |
| TCGA-DU-7013 | -1077.88419 | -147.563966 | -1225.448156 | 0.910995175 |
| TCGA-QH-A6X5 | -1454.665458 | -734.1206451 | -2188.786103 | 0.960026685 |
| TCGA-HT-7688 | -1559.926242 | -1332.35341 | -2892.279652 | 0.983765883 |
| TCGA-DU-8163 | -891.2849513 | 348.7345013 | -542.55045 | 0.865149924 |
| TCGA-R8-A6MK | -1631.211839 | -776.7727503 | -2407.984589 | 0.968534503 |
| TCGA-DU-A6S3 | -1683.323057 | -1358.243479 | -3041.566536 | 0.987461911 |
| TCGA-DU-A5TS | -1279.916743 | -362.6334171 | -1642.55016 | 0.934522997 |
| TCGA-HT-7611 | -1012.506497 | -127.3949593 | -1139.901457 | 0.905744658 |
| TCGA-DU-7009 | -1770.22993 | -1435.678348 | -3205.908278 | 0.990982302 |
| TCGA-TM-A84L | -828.0495669 | 152.819725 | -675.2298419 | 0.874752604 |
| TCGA-FG-A711 | -1698.0054 | -648.2325743 | -2346.237974 | 0.966238987 |
| TCGA-HT-7616 | -550.2962649 | 761.0333566 | 210.7370917 | 0.804525061 |
| TCGA-DB-A64R | -1755.917013 | -1354.473887 | -3110.3909 | 0.989006267 |
| TCGA-E1-A7Z6 | -1278.792791 | -440.7273658 | -1719.520156 | 0.938484362 |
| TCGA-S9-A6WD | -1771.445395 | -1114.215959 | -2885.661355 | 0.983591079 |
| TCGA-QH-A870 | -1614.301843 | -840.4792827 | -2454.781125 | 0.970221238 |
| TCGA-FG-8188 | -1061.84792 | -361.6099628 | -1423.457883 | 0.922595827 |
| TCGA-DU-5853 | -960.4008328 | 327.3614008 | -633.0394319 | 0.871734877 |
| TCGA-S9-A6TY | -1977.602931 | -1560.196742 | -3537.799673 | 0.996331778 |
| TCGA-E1-5318 | -1055.92643 | -799.5315003 | -1855.45793 | 0.945187656 |
| TCGA-DU-7309 | -1045.080857 | -686.2460619 | -1731.326919 | 0.939081428 |
| TCGA-HT-A614 | -1391.017384 | -488.2849537 | -1879.302337 | 0.946324737 |
| TCGA-FG-8187 | -1209.968078 | -555.702952 | -1765.67103 | 0.940802162 |
| TCGA-S9-A6TX | -1408.249842 | -718.8998574 | -2127.149699 | 0.957454952 |
| TCGA-HT-A74H | -416.624971 | 206.0692283 | -210.5557428 | 0.839692242 |
| TCGA-TQ-A7RG | -1353.953424 | -486.5927897 | -1840.546214 | 0.944470666 |
| TCGA-DB-A4XE | -1565.334213 | -688.9493795 | -2254.283593 | 0.96267339 |
| TCGA-S9-A7R8 | -1160.291721 | 72.31380332 | -1087.977917 | 0.902488093 |
| TCGA-HT-7875 | -1700.967115 | -1389.841143 | -3090.808259 | 0.988577118 |
| TCGA-HT-7481 | -1460.848502 | -1149.513112 | -2610.361614 | 0.975499448 |
| TCGA-HT-7677 | -1480.400931 | -942.8979543 | -2423.298886 | 0.969091526 |
| TCGA-HT-A5R9 | -1508.226818 | -1164.636261 | -2672.863079 | 0.977476771 |
| TCGA-FG-A87Q | -230.2658685 | 785.6504374 | 555.3845689 | 0.773462059 |
| TCGA-S9-A6U5 | -1320.641422 | 201.7127536 | -1118.928669 | 0.904435601 |
| TCGA-HT-A74J | -1444.224143 | -364.1241152 | -1808.348259 | 0.942907086 |
| TCGA-QH-A6CZ | -1670.079123 | -1317.78069 | -2987.859813 | 0.986186766 |
| TCGA-WY-A85D | -1311.383565 | -170.824242 | -1482.207807 | 0.925888268 |
| TCGA-S9-A7IQ | -1569.977127 | -1654.534739 | -3224.511867 | 0.991344513 |
| TCGA-DU-A76O | -1028.858144 | 611.1521732 | -417.7059707 | 0.855814576 |
| TCGA-HT-A5R5 | -743.4117243 | 469.8206733 | -273.5910509 | 0.844681103 |
| TCGA-VM-A8CB | -1859.620424 | -1019.271101 | -2878.891524 | 0.983411312 |
| TCGA-HT-8108 | -1146.080849 | 346.3351232 | -799.7457262 | 0.88346272 |
| TCGA-E1-A7YW | -1359.508344 | -296.3316073 | -1655.839951 | 0.935215509 |
| TCGA-S9-A6WI | -1271.051615 | -1328.519617 | -2599.571233 | 0.975149763 |
| TCGA-HT-7469 | -1449.719248 | -864.5692096 | -2314.288458 | 0.965020046 |
| TCGA-06-0138 | 142.7950444 | 1077.654167 | 1220.449211 | 0.707999416 |
| TCGA-12-3653 | -960.0480206 | 424.9164502 | -535.1315704 | 0.86460326 |
| TCGA-32-1982 | 132.4227748 | 1191.791371 | 1324.214145 | 0.697161031 |
| TCGA-02-2483 | -528.589729 | 413.6911585 | -114.8985704 | 0.831984378 |
| TCGA-14-1829 | 96.04960589 | 1027.812878 | 1123.862484 | 0.717940479 |
| TCGA-06-0238 | -309.7555022 | 888.1267499 | 578.3712476 | 0.771318962 |
| TCGA-28-2513 | 376.3048801 | 1468.224311 | 1844.529191 | 0.640426634 |
| TCGA-27-2521 | -1071.548519 | -590.188778 | -1661.737297 | 0.935521671 |
| TCGA-02-2485 | -849.8647047 | -173.9710068 | -1023.835712 | 0.898392828 |
| TCGA-06-2558 | -236.3346371 | 284.4423609 | 48.10772387 | 0.818472574 |
| TCGA-06-5413 | 211.7466582 | 939.0037301 | 1150.750388 | 0.715187487 |
| TCGA-19-0957 | -345.9084686 | 495.9275854 | 150.0191167 | 0.809786453 |
| TCGA-19-1787 | -526.1582999 | 600.7787174 | 74.6204175 | 0.816230391 |
| TCGA-06-1804 | -652.6227352 | -99.49970786 | -752.1224431 | 0.88016608 |
| TCGA-06-5416 | -1252.127023 | -732.5895747 | -1984.716598 | 0.951212584 |
| TCGA-26-5133 | -1018.327849 | -674.9173945 | -1693.245244 | 0.93714553 |
| TCGA-06-0130 | 1019.927507 | 2395.770375 | 3415.697882 | 0.447908152 |
| TCGA-27-2528 | -401.6903422 | -669.9699037 | -1071.660246 | 0.901453838 |
| TCGA-12-1597 | -702.9050259 | 191.3482333 | -511.5567927 | 0.862859339 |
| TCGA-06-0187 | -483.2466869 | 627.4240614 | 144.1773745 | 0.810289272 |
| TCGA-06-0178 | -463.6953506 | 725.3150702 | 261.6197196 | 0.800066695 |
| TCGA-27-2523 | -1172.223942 | -287.5810583 | -1459.805001 | 0.924640888 |
| TCGA-06-0141 | 308.5662292 | 1702.970956 | 2011.537185 | 0.62140822 |
| TCGA-28-5220 | -694.7989111 | 562.5011412 | -132.29777 | 0.833398621 |
| TCGA-28-2514 | -857.4404448 | -162.4562222 | -1019.896667 | 0.898138734 |
| TCGA-28-5204 | -760.7509978 | 227.8889766 | -532.8620211 | 0.864435823 |
| TCGA-28-5218 | 936.7054522 | 1570.02355 | 2506.729003 | 0.562867017 |
| TCGA-06-0168 | 189.2656211 | 1163.433035 | 1352.698656 | 0.694157385 |
| TCGA-06-2570 | -472.8951139 | 744.1313334 | 271.2362195 | 0.79921907 |
| TCGA-32-2638 | 22.58775976 | 918.8105457 | 941.3983054 | 0.736324955 |
| TCGA-19-1390 | -1436.311867 | -559.0631694 | -1995.375037 | 0.951694135 |
